# Whole-body irradiation causes long-term impairments in the maintenance and function of naïve CD4 T cells specific for self- and non-self-antigens

**DOI:** 10.1093/jimmun/vkag174

**Published:** 2026-07-13

**Authors:** Shravan Kumar Kannan, Ti-Ara J Turner, Mohammad Heidarian, Madison R Mix, Caleb Y Kim, Cori Fain, Shailesh K Shahi, Elizabeth A Escue, Ifechukwu Ezeilo, Thomas S Griffith, Ashutosh K Mangalam, John T Harty, Vladimir P Badovinac

**Affiliations:** Interdisciplinary Graduate Program in Immunology, Carver College of Medicine, University of Iowa, Iowa City, IA, United States; Department of Pathology, Carver College of Medicine, University of Iowa, Iowa City, IA, United States; Holden Comprehensive Cancer Center, Carver College of Medicine, University of Iowa, Iowa City, IA, United States; Interdisciplinary Graduate Program in Immunology, Carver College of Medicine, University of Iowa, Iowa City, IA, United States; Department of Pathology, Carver College of Medicine, University of Iowa, Iowa City, IA, United States; Holden Comprehensive Cancer Center, Carver College of Medicine, University of Iowa, Iowa City, IA, United States; Department of Pathology, Carver College of Medicine, University of Iowa, Iowa City, IA, United States; Holden Comprehensive Cancer Center, Carver College of Medicine, University of Iowa, Iowa City, IA, United States; Experimental Pathology Graduate Program, Carver College of Medicine, University of Iowa, Iowa City, IA, United States; Interdisciplinary Graduate Program in Immunology, Carver College of Medicine, University of Iowa, Iowa City, IA, United States; Department of Pathology, Carver College of Medicine, University of Iowa, Iowa City, IA, United States; Holden Comprehensive Cancer Center, Carver College of Medicine, University of Iowa, Iowa City, IA, United States; Medical Scientist Training Program, Carver College of Medicine, University of Iowa, Iowa City, IA, United States; Center for Immunology, University of Minnesota, Minneapolis, MN, United States; Microbiology, Immunology, and Cancer Biology Program, University of Minnesota, Minneapolis, MN, United States; Department of Pathology, Carver College of Medicine, University of Iowa, Iowa City, IA, United States; Holden Comprehensive Cancer Center, Carver College of Medicine, University of Iowa, Iowa City, IA, United States; Department of Pathology, Carver College of Medicine, University of Iowa, Iowa City, IA, United States; Holden Comprehensive Cancer Center, Carver College of Medicine, University of Iowa, Iowa City, IA, United States; Experimental Pathology Graduate Program, Carver College of Medicine, University of Iowa, Iowa City, IA, United States; Department of Pathology, Carver College of Medicine, University of Iowa, Iowa City, IA, United States; Holden Comprehensive Cancer Center, Carver College of Medicine, University of Iowa, Iowa City, IA, United States; Experimental Pathology Graduate Program, Carver College of Medicine, University of Iowa, Iowa City, IA, United States; Interdisciplinary Graduate Program in Immunology, Carver College of Medicine, University of Iowa, Iowa City, IA, United States; Department of Pathology, Carver College of Medicine, University of Iowa, Iowa City, IA, United States; Holden Comprehensive Cancer Center, Carver College of Medicine, University of Iowa, Iowa City, IA, United States; Center for Immunology, University of Minnesota, Minneapolis, MN, United States; Microbiology, Immunology, and Cancer Biology Program, University of Minnesota, Minneapolis, MN, United States; Interdisciplinary Graduate Program in Immunology, Carver College of Medicine, University of Iowa, Iowa City, IA, United States; Department of Pathology, Carver College of Medicine, University of Iowa, Iowa City, IA, United States; Holden Comprehensive Cancer Center, Carver College of Medicine, University of Iowa, Iowa City, IA, United States; Experimental Pathology Graduate Program, Carver College of Medicine, University of Iowa, Iowa City, IA, United States; Medical Scientist Training Program, Carver College of Medicine, University of Iowa, Iowa City, IA, United States; Interdisciplinary Graduate Program in Immunology, Carver College of Medicine, University of Iowa, Iowa City, IA, United States; Department of Pathology, Carver College of Medicine, University of Iowa, Iowa City, IA, United States; Experimental Pathology Graduate Program, Carver College of Medicine, University of Iowa, Iowa City, IA, United States; Medical Scientist Training Program, Carver College of Medicine, University of Iowa, Iowa City, IA, United States; Interdisciplinary Graduate Program in Immunology, Carver College of Medicine, University of Iowa, Iowa City, IA, United States; Department of Pathology, Carver College of Medicine, University of Iowa, Iowa City, IA, United States; Holden Comprehensive Cancer Center, Carver College of Medicine, University of Iowa, Iowa City, IA, United States; Experimental Pathology Graduate Program, Carver College of Medicine, University of Iowa, Iowa City, IA, United States; Medical Scientist Training Program, Carver College of Medicine, University of Iowa, Iowa City, IA, United States

**Keywords:** brain CD4 T_RM_, CD4 T cells, EAE, intracellular infections, irradiation

## Abstract

Whole-body irradiation (WBI) consistently induces radiation-associated lymphopenia, a complication linked to poor prognosis and reduced overall survival. Understanding the quantitative and qualitative changes in naïve CD4 T cells after WBI is critical because these cells play key roles in both host defense and the propagation of autoimmunity. Here, we show that WBI triggers a rapid decline in the naïve CD4 T-cell pool, followed by thymus-dependent numerical recovery. Shortly after WBI, mice exhibit a reduced frequency of lymphocytic choriomeningitis virus (LCMV) epitope GP_66-77_–specific naïve CD4 T-cell precursors. Upon infection with LCMV, this reduction leads to delayed effector expansion, impaired memory differentiation, and diminished cytokine production. WBI also disrupts blood–brain barrier (BBB) integrity, enhancing infiltration of GP_66-77_–specific memory CD4 T cells into the brain and promoting enrichment of brain-resident tissue-resident memory CD4 T cells. Similarly, encephalitogenic MOG_38-49_–specific CD4 T-cell precursors are reduced after WBI, resulting in delayed but exacerbated experimental autoimmune encephalomyelitis (EAE) compared with nonirradiated controls. WBI-mediated BBB disruption facilitates the entry of encephalitogenic MOG_38-49_–specific CD4 T cells into the spinal cord, promoting EAE induction and progression even in the absence of pertussis toxin administration. Together, these findings highlight both immune-intrinsic and -extrinsic effects of WBI in shaping naïve CD4 T-cell responses in the context of infection and autoimmunity.

## Graphical abstract

**Graphical Abstract:**
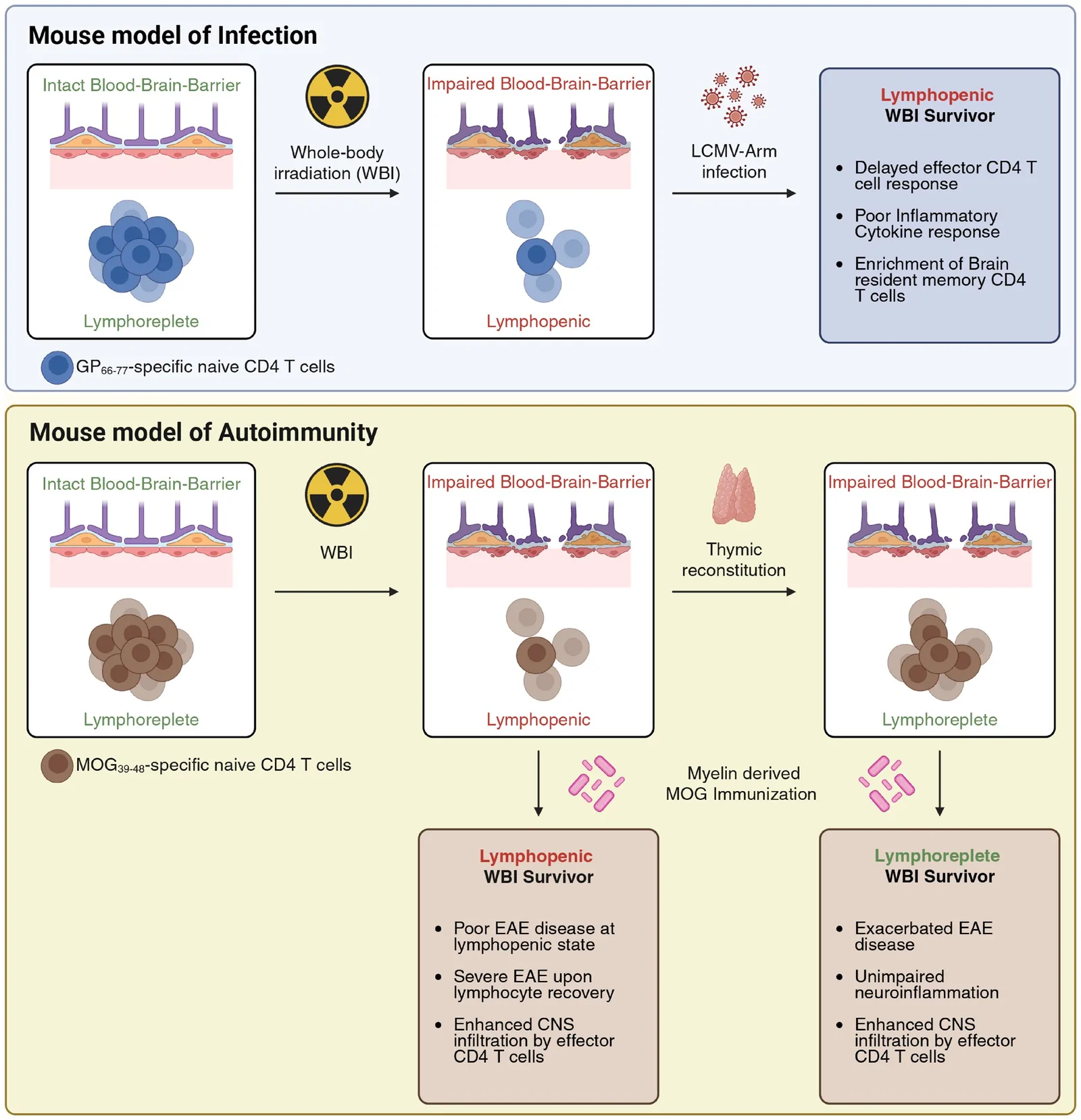
Created in BioRender. Kannan, S. K. (2026) https://BioRender.com/1bjd002

## Introduction

There is a global push to triple nuclear energy production across countries by 2050 to meet growing electricity demand while maintaining the countries’ commitment to environmental sustainability. Significant investments are being directed toward expanding nuclear access through the deployment of small modular reactors and microreactors, which can generate energy from shipping-container-sized units. Historically, the most fatal and life-threatening radiation exposure to humans has been from nuclear bombs and accidents at nuclear facilities. Although such exposures are less likely, the most common exposures come from radiation therapy (RT) to treat solid tumors and hematologic malignancies. RT improves cancer prognosis directly by causing DNA damage-mediated tumor regression and indirectly by improving the immune response by generating tumor neo-antigens. These RT approaches carry the risk of adverse side effects because they also affect healthy tissues, particularly immune cells.^1^^,^^2^ Retrospective clinical studies testing immunotherapy efficacy show that RT-mediated lymphopenia is very common among patients with cancer and is associated with poor progression-free and overall survival.^3^ Therefore, understanding the maintenance and functional potential of T cells after radiation exposure is imperative to developing effective medical countermeasures.

Upon infection or vaccination, naïve CD4 and CD8 T cells differentiate into effector and memory populations, providing long-term protection to future pathogen encounters.^4^^,^^5^ Although CD8 T-cell responses are key to curtailing intracellular pathogens and tumor cells, CD4 T cells can provide broader protection against both intra- and extracellular pathogens.^6–9^ CD4 CTLs play key roles in clearing chronic viral infections and, recently, in solid tumors, particularly in compensating for exhausted CD8 T-cell responses.^10–13^ CD4 T-cell help is also critical for establishing long-lasting, productive CD8 T-cell and B-cell responses against infections.^14–17^ Although pathogen-derived epitopes promote protective CD4 T-cell responses, TCR recognition of self-antigens can expand autoreactive effector cells, leading to autoimmune diseases. Autoreactive effector CD4 T cells are responsible for inflammation and immunopathology of multiple sclerosis (MS), rheumatoid arthritis, and systemic lupus erythematosus.^18–20^ Because CD4 T cells can be pathogenic or protective, depending on self- or non–self-epitope-mediated TCR activation, it is important to understand the effects of radiation exposure on their maintenance and function.

Existing studies address the effect of radiation exposure on naïve T-cell recovery but have not provided mechanistic insights into their functional response to infection and autoimmunity. RT, if not directly implicated, has been associated with increased autoimmune pathology and poor protection from infections. For example, RT is the standard of care for glioblastoma, yet 42% of patients without a prior history of MS develop this autoimmune disorder within 1 year of initiating RT.^21^ Similarly, whole-body irradiation (WBI), a conditioning regimen prior to hematopoietic stem cell transplantation (HSCT), can damage transplanted HSCs through radiation-induced bystander effects, thereby increasing susceptibility to infections.^22^^,^^23^ In addition to direct effects on immune cells, WBI induces systemic immune-extrinsic changes, including cardiac dysfunction, gut dysbiosis, tissue inflammation, and compromised barrier integrity.^1^^,^^24–26^ Given their plasticity and sensitivity to environmental cues, CD4 T cells are particularly susceptible to these extrinsic changes, which may affect their maintenance and function.^27^

Here, we define how WBI affects CD4 T-cell intrinsic function and the extrinsic tissue environment to regulate CD4 T-cell responses. Using mouse models of viral infection (lymphocytic choriomeningitis virus [LCMV]), bacterial infection (*Listeria monocytogenes*), and autoimmunity (experimental autoimmune encephalomyelitis [EAE]), we demonstrate that WBI-mediated reduction in lymphocyte precursors and tissue damage synergistically work to regulate CD4 T-cell responses.

## Materials and methods

### Sex as a variable

In all the experiments involving mouse models of irradiation and infection, both male and female mice were used, with no differences observed in the kinetics of loss, recovery, and phenotypic changes to the bulk naive CD4^+^ T cells after WBI. Thereafter, our experimental designs, involving thymectomized mice and MHC class II tetramer-based enrichment, used female mice, with the findings expected to be relevant for both sexes. For all the experiments involving mouse models of EAE, sex was not analyzed as a variable. Female mice were used for EAE based on literature indicating more robust demyelination, larger immune-cell infiltration, and more reproducible disease severity in female mice compared with male mice.^28^

### Mice

Inbred C57BL/6 mice were purchased from Charles River and maintained in the animal facilities at the University of Iowa at the appropriate biosafety level. We used 6- to 10-wk old mice for all experiments. Outbred Swiss Webster mice, 6- to 8-wk old, were purchased from Charles River. And 6- to 8-wk old TCR-tg 2D2 mice were purchased from The Jackson Laboratory and bred with Thy1.2^2+^ wild-type (WT) C57BL/6 mice to generate Thy1.1^+^1.2^+^ 2D2 donors. Thymectomized WT C57BL/6 mice were purchased from The Jackson Laboratory, where mice underwent thymectomy around 6 weeks of age.

### Irradiation

WBI was performed using the 225-kVp rotating X-ray tube (Small Animal Radiation Research platform; Xstrahl, Atlanta, GA) radiation cabinet available at the Radiation Core at the University of Iowa. The cabinet releases a precalibrated dose per minute, and the Core uses the MuriPlan software to calculate the duration of exposure needed to achieve the desired overall dose. For irradiation experiments, 1 Gy, 5 Gy, or 10 Gy radiation doses were used. Mice that received 5 Gy of WBI (hereafter, 5 Gy WBI mice) were placed into mouse pie cages and were exposed to a 5 Gy dose for ∼8 min. Mock-treated (0 Gy) mice were kept in the mouse pie cages inside the cabinet without radiation exposure for the same duration as the highest dose group (5 Gy or 10 Gy) of each experiment.

### Enrichment and characterization of epitope-specific naïve CD4± T cells

A tetramer-based enrichment protocol using GP_66-77_ and MOG_38–49_ containing I-A^b^ MHC II tetramers was used.^29–31^ In brief, splenocytes were stained with PE-conjugated pMHC II tetramers in 200 µL of tetramer staining buffer (PBS containing 5% FBS, 2 mM EDTA, 1:50 normal mouse serum, and 1:200 anti-CD16/32 mAb). The cells were incubated in the dark at room temperature for 1 h, followed by a wash in 5 mL of ice-cold FACS buffer. The tetramer-stained cells were then resuspended in 500 µL of FACS buffer, mixed with 6.25 µL of anti-PE (EasySep) Ab cocktail, and incubated at room temperature in the dark for 15 min. Magnetic particles (50 μL of beads per sample; EasySep positive selection kit II) were added and incubated at room temperature in the dark for 10 min. The cells were washed, resuspended in 2.5 mL cold FACS buffer, and passed through an EasySep Magnet (StemCell Technologies) to yield the enriched tetramer-positive population. The resulting enriched fractions were stained with a cocktail of fluorochrome-labeled mAb containing Thy1.2, CD4, CD8, CD44, and “dump/live” (CD11b, CD11c, B220, F4/80, Live/Dead). Cell numbers for each sample were determined using AccuCheck Counting Beads (Invitrogen). Samples were then analyzed using a Fortessa flow cytometer (BD) and FlowJo software (Tree Star, Ashland, OR).

### Infections

Mice were infected with 10^6^ CFU of *actA plus InlB*-deficient gp_33_+gp_61_ expressing attenuated *L. monocytogenes* (a.LM) (i.v.), 2 × 10^6^ PFU of LCMV-Clone 13 (i.p.), or 2 × 10^5^ PFU of LCMV-Armstrong (i.p.).

### EAE disease induction and evaluation

EAE was induced and evaluated as described previously.^32^ Briefly, C57BL/6 mice were immunized s.c. in both flanks with 100 µg of MOG_35-55_ peptides (GenScript) that were emulsified in complete Freund’s adjuvant containing *Mycobacterium tuberculosis* H37Ra (100 µg/mouse; Becton Dickinson and Company, Sparks, MD). Pertussis toxin (PTX**)** (80 ng/mouse; Sigma Chemicals, St. Louis, MO) was administered i.p. on days 0 and 2 after immunization. In certain experiments, PTX was not administered on both days. For experiments involving 2D2 T cells, 5,000 TCR-tg–naïve 2D2 cells (Vα3.2^+^Vβ11^+^; Thy1.1^+^Thy1.2^+^) were adoptively transferred (i.v.) into each mouse, 1 d before immunization. Disease severity was scored using a standard 0 to 5 scale^32^^,^^33^ as follows: 0, no clinical symptoms; 1, loss of tail tonicity; 2, hind limb weakness; 3, hind limb paralysis; 4, fore limb weakness; 5, moribund or death.

### Tissue processing and cell isolation for flow cytometry

Peripheral blood was collected through the retroorbital sinus. Single-cell suspensions from spleen and lymph nodes were generated after mashing the tissue through a 70-µm cell strainer without enzymatic digestion. Ammonium–chloride–potassium lysis buffer was used for RBC lysis from peripheral blood and spleen samples. In experiments involving the salivary gland and brain harvest, mice received an i.v. injection of 2 µg of anti-CD45.2 (30-F11, BioLegend) conjugated to a fluorophore 3 min prior to euthanasia. Both the salivary gland (submandibular region) tissues were isolated while avoiding the cervical lymph nodes, chopped, and incubated in 40% liver digest medium (Gibco, 17703034; made with RPMI-1640 medium plus 10% FBS [RP10]) for 1 h, shaking at 120 rpm at 37 °C. The dissociated tissue was then passed through a 70-µm filter and washed once with cold RP10 before proceeding with counting and staining.

Brain tissue was isolated and digested in Collagenase D/DNase for 45 min at 37 °C. Brain tissue was then dissociated through a 70-µm filter and separated using a layered 70% and 37% Percoll gradient spun at 913 xg for 20 min at 25 °C without brake. Brain mononuclear cells were collected at the gradient interface. In experiments involving spinal cord (SC) harvest, mice were anesthetized with CO_2_ and were quickly perfused through the left ventricle with cold PBS. SCs were flushed through the vertebral column with cold RPMI medium. The length of the SC was measured using a ruler. Upon homogenizing, the cells were separated using a layered 80% and 40% Percoll gradient spun at 2,000 RPM for 30 min at 25 °C without brakes. SC leukocyte cells were collected at the gradient interface.

### Cell staining and flow cytometry

To assess Ag-specific CD4 T-cell populations, single-cell suspensions were incubated with 50 µL of Fc block (1:100) at 4 °C for 15 min. The cells were incubated with 50 µL of PE-conjugated GP_66-77_:I-A^b^ or MOG_38–49_:I-A^b^ tetramers (final concentration of 4 to 6 µg/mL; courtesy of the National Institutes of Health Tetramer Core Facility) at 37 °C for 30 min. The cells were washed and then subjected to surface staining using a cocktail of fluorescently labelled mAbs with 1:100 Fc block at 4 °C for 20 to 30 min and then fixed using Cytofix (BD Biosciences). To measure cytokine production after infection, 10^6^ splenocytes were plated in the absence or presence of 0.05 or 5 ng/mL GP_61-80_ peptide with Brefeldin A (BD Biosciences) for 5 h at 37 °C. Cells were washed, stained for surface markers, and then permeabilized using Cytofix/Cytoperm (BD Biosciences) for 15 min. After washing, they were incubated with cytokine-specific mAb in PERM wash at 4 °C for 30 to 60 min.

To measure Ki67 expression and cytokine production after EAE, cells from the SC were isolated as described earlier. SC cells from 2 mice with the same disease score within each group were pooled into a single sample; ie cells from six 0 Gy and twenty 5 Gy mice became 3 and 10 biological replicates, respectively. These cells were then plated in the presence of Brefeldin A (BD Biosciences) for 1 h at 37 °C. After washing, they were stained with the MOG_38-49_:I-A^b^ tetramers and cell surface markers as described above. They were then incubated in the fixation/permeabilization buffer (eBioscience FoxP3/Transcription factor staining buffer kit) for 45 min at 4 °C. Upon washing, these cells were stained with Ki67 and other intracellular cytokine markers for 45 min at 4 °C. Data were acquired using Cytek Aurora (Cytek Biosciences, Fremont, CA) and analyzed using FlowJo software version 10 (Tree Star, Ashland, OR).

### Brain-tissue sectioning and microscopy

Mice were injected i.v. with 66 kDa FITC-conjugated albumin 1 h prior to euthanasia. Brain tissue was harvested without perfusion, fixed in 4% paraformaldehyde overnight, and rinsed twice with PBS. Whole brains were submerged in 4% low-melting point agarose (Promega) within Peel-A-Way embedding molds (Thermo Fisher) and held at 4 °C to solidify. Embedded brain tissue was then sectioned in 100 to 200 µm sections using a Pelco easiSlicer vibratome (Ted Pella, Inc). Sectioned brain tissue was washed twice in PBS, fixed in 4% paraformaldehyde, washed again in PBS, and mounted on Superfrost Plus microscope slides (Fisher). After drying, slide coverslips were placed with Prolong Gold Antifade Mountant with DAPI (ThermoFisher). Whole-slide images were acquired using a slide-scanning microscope (Olympus VS120) and reviewed via OlyVIA software (Olympus).

### Quantification and statistical analysis

All statistical analyses were performed using GraphPad Prism (versions 9.0–10.4). Fold changes were calculated by dividing the means of the groups specified. When indicated, 2-tailed unpaired Student’s *t* tests were performed when comparing 2 independent groups, 1-way ANOVA with Tukey’s multiple comparisons test when comparing more than 2 groups, and 2-way ANOVA with Šidák’s or Dunnett’s multiple comparisons test when comparing 2 or more groups, respectively, for more than 1 variable. *P* values are indicated in individual figures or figure legends.

### Study approval

Experimental procedures using mice were approved by the University of Iowa Animal Care and Use Committee under protocol 2121915.

## Results

### WBI induces a dose-dependent, rapid, and severe decline in naïve CD4 T-cell numbers, followed by gradual recovery over time

**Figure 1:**
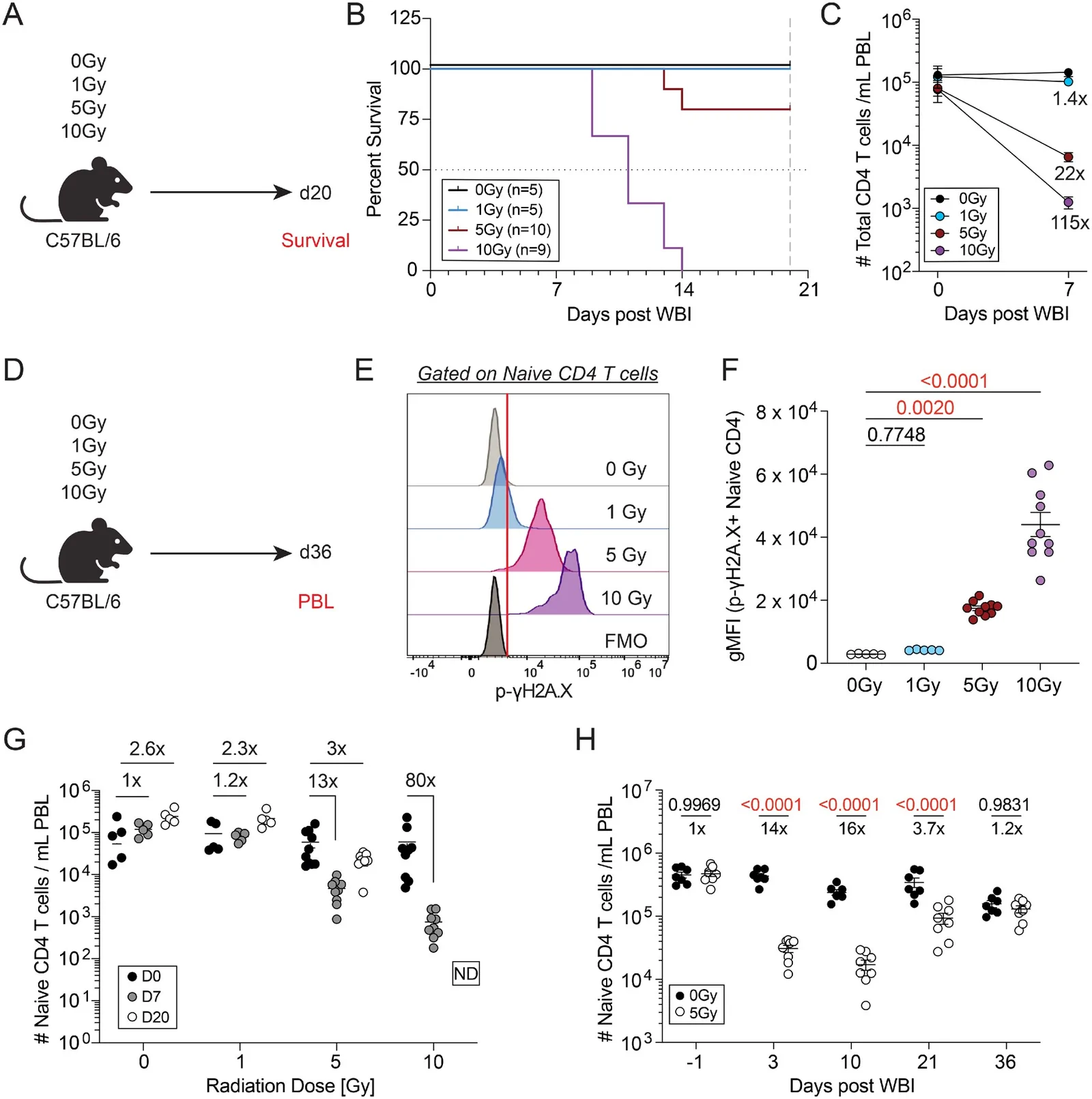
WBI induces a dose-dependent and transient decline in naïve CD4 T-cell numbers. (A and B) Experimental design: Mice subjected to different doses of WBI were monitored for survival over time. (C) Number of total CD4 T cells per mL of blood up to 7 dpWBI. (D–F) Experimental design: Blood was collected 3 h after WBI and stained for p-γH2A.X expression in naive CD4 T cells. (E) Representative flow plots show the geometric mean fluorescence intensity (gMFI) of p-γH2A.X expression in naïve (CD44^−^) CD4 T cells. (F) p-γH2A.X gMFI in naïve (CD44^−^) CD4 T cells from mice that received from 0 to 10 Gy WBI. Statistical analyses were performed using 1-way ANOVA with Šidák’s multiple comparisons test. (G) Longitudinal assessment of the number of (CD44^−^) naïve CD4 T cells in the blood of WBI mice. The fold change for the same host before and after WBI is highlighted. (H) Number of naïve (CD49d^lo^) CD4 T cells in the blood of mice that received 0 Gy or 5 Gy WBI over 36 dpWBI. *P* values were derived from 2-way ANOVA with Šidák’s multiple comparisons test. Data in (A–G) are representative of 2 independent experiments, and those in (H) are of 3 independent experiments with 5 to 10 mice per group. (C and H) The fold change between the WBI host and the 0 Gy host at specified days is highlighted. All graphs show the mean ± SEM.Graphical illustrations (A and D) are created with BioRender.com (http://biorender.com).

There is substantial evidence linking WBI dose to health condition severity, but the effect of WBI dose on naïve CD4 T cells is not fully characterized.^34^^,^^35^ To examine how WBI affects naïve CD4 T-cell number and function, we first subjected naïve specific pathogen–free inbred C57BL/6 (B6) mice to either 0 Gy, 1 Gy, 5 Gy, or 10 Gy doses of WBI (Fig. 1A). Whereas 100% of mice receiving 0 Gy or 1 Gy WBI survived 20 d (Fig. 1B), 100% mortality was observed in the 10 Gy group within 2 wk, and 20% of mice in this experiment succumbed after the 5 Gy dose. Seven days after WBI (dpWBI), we observed a WBI dose-dependent decline in total CD4 T-cell numbers in the blood, with 5 Gy and 10 Gy exposures resulting in severe reductions—approximately 22-fold and 115-fold, respectively—in total CD4 T cells per milliliter of blood compared with unirradiated (0 Gy) controls (Fig. 1C).

DNA damage is a major driver of ionizing radiation–mediated cell death.^36^ When a cell sustains DNA damage, it leads to a transient increase in the phosphorylation of histone γH2A.X at serine 139, which signals DNA damage to initiate DNA repair mechanisms.^37^^,^^38^ To assess the extent to which the WBI dose–dependent decline in CD4 T cells was driven by DNA damage, peripheral blood leukocytes were collected 3 h after irradiation and stained for γH2A.X (p-Ser139), along with CD4 T-cell markers for flow cytometric analysis (Fig. 1D). Naïve (CD44^−^) CD4 T cells exhibited a WBI dose-dependent increase in the expression of γH2A.X (p-Ser139) (Fig. 1E and F). Similar WBI-induced DNA damage was observed in outbred Swiss Webster mice, in which naïve CD4 T cells were reduced ∼38-fold by 7 dpWBI (Fig. S1). Overall, these data suggest that DNA damage drives the WBI dose-dependent decline in naïve CD4 T cells in inbred and outbred mice.

Next, to determine the extent of numeric recovery of (CD44^−^) naïve CD4 T cells after WBI, we tracked their numbers in the blood over time following increasing doses of WBI (Fig. 1D and Fig. S2A). Mice exposed to 10 Gy WBI had an ∼80-fold reduction in naïve CD4 T-cell numbers by 7 dpWBI and did not survive to 20 dpWBI (Fig. 1G). In contrast, mice receiving 5 Gy WBI experienced an ∼13-fold reduction by 7 dpWBI, with partial recovery by 20 dpWBI, as indicated by only a ∼3-fold decrease relative to baseline (Fig. 1G). The number of naïve CD4 T cells in 1 Gy WBI mice remained indistinguishable from those in 0 Gy controls throughout the 20-d observation period (Fig. 1G).

Together, these data show that 5 Gy WBI is a sublethal dose causing early lymphopenia followed by gradual recovery. Based on these findings, we selected 5 Gy for subsequent experiments to assess its effect on naïve CD4 T-cell function. To determine how long naïve CD4 T cells take to recover to similar levels as 0 Gy controls, we tracked their numbers (CD49d^lo^) (Fig. S2B) in blood over time. Here, complete recovery occurred after 36 dpWBI (Fig. 1H). Overall, these findings demonstrate a WBI dose-dependent DNA damage-mediated decline in naïve CD4 T-cell numbers, followed by gradual recovery over time.

### Recovery of naïve CD4 T cells after WBI relies on thymic output

**Figure 2:**
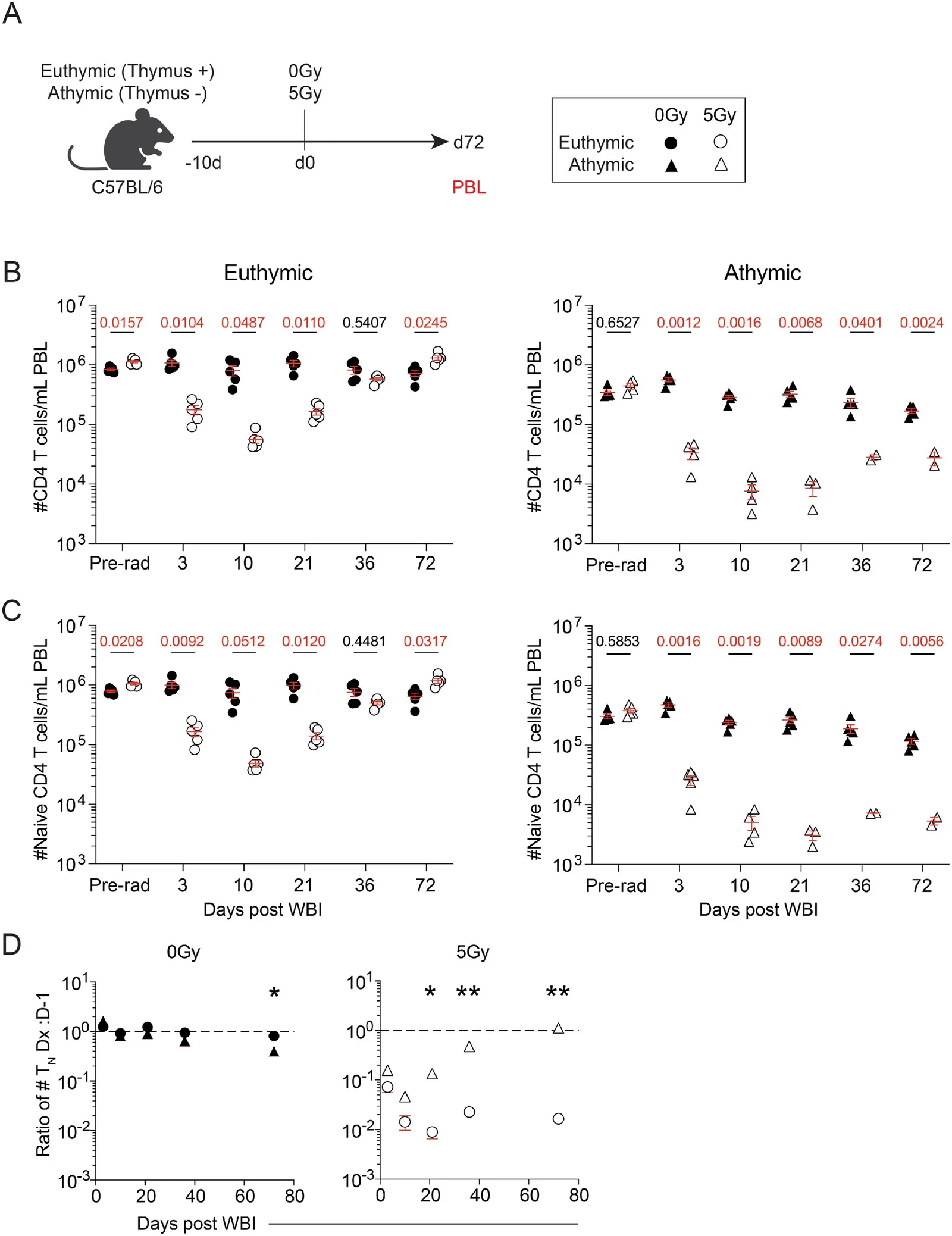
Thymic output is critical for the timely recovery of naïve CD4 T cells after WBI. (A) Experimental design: Euthymic and athymic mice were subjected to 0 Gy or 5 Gy WBI, and longitudinal cellular analyses were performed in the blood. (B and C) Number of total CD4 T cells (B) and naïve CD4 T cells (C) per milliliter of blood among euthymic (left) and athymic (right) mice at the indicated time points. (D) The number of naïve CD4 T cells at each indicated time point was divided by the pre-WBI (the day before WBI [D-1]) number from the same mouse, and the means of this ratio were plotted for euthymic and athymic mice that received 0 Gy (left) and 5 Gy (right) dose of WBI. Data are representative of 2 independent experiments with 2 to 5 mice per group. Statistical significance was determined by 2-way ANOVA with Šidák’s or mixed-effects model with Geisser-Greenhouse correction. *P ≤ 0.05, **P ≤ 0.01. All graphs show the mean ± SEM. Graphical illustrations (A) are created with BioRender.com (http://biorender.com).

The recovery of naïve CD4 T cells observed earlier (Fig. 1H) is potentially driven by thymic reconstitution combined with homeostatic proliferation of surviving naïve CD4 T cells after WBI. Consequently, we tested whether the naïve CD4 T cells that survive after WBI can reconstitute the naïve CD4 T-cell compartment. To that end, we obtained thymectomized B6 mice (athymic) that had their thymi removed at age 6 wk. We also obtained age-matched WT (euthymic) mice, which would allow us to assess the contribution of new thymic emigrants after WBI to naïve CD4 T-cell reconstitution. The euthymic and athymic mice were exposed to 0 Gy (mock) or 5 Gy doses of WBI, and the number of naïve CD4 T cells in the blood was assessed over time (Fig. 2A). Consistent with data in Fig. 1, numbers of naïve CD4 T cells in euthymic mice declined significantly after 10 dpWBI and recovered to the same level as the 0 Gy group by 36 dpWBI (Fig. 2B–D). However, the number of naïve CD4 T cells in athymic mice remained lower for up to 72 dpWBI compared with the 0 Gy group. These data show that the naïve CD4 T cells that survive radiation exposure cannot restore the naïve CD4 T-cell compartment without thymic production.

### Rapid reduction in naïve CD4 T-cell precursors after WBI consequently limits effector expansion in response to intracellular infections

During any infection, cognate Ag recognition and activation of naïve CD4 T cells drive their expansion in numbers and acquisition of effector function.^39^^,^^40^ The broad epitope specificity of the naïve T-cell compartment enables protection against diverse pathogens.^23^^,^^41^^,^^42^ As a consequence, precursor frequencies for any single antigenic epitope are low,^43^ making functional studies of Ag-specific naïve CD4 T cells challenging. One approach to overcome this limitation is the peptide:MHC-II (pMHC-II) tetramer-based enrichment, which enables the identification, quantification, and phenotype assessment of naïve (Ag nonexperienced) epitope-specific CD4 T cells from mouse splenocytes.

**Figure 3:**
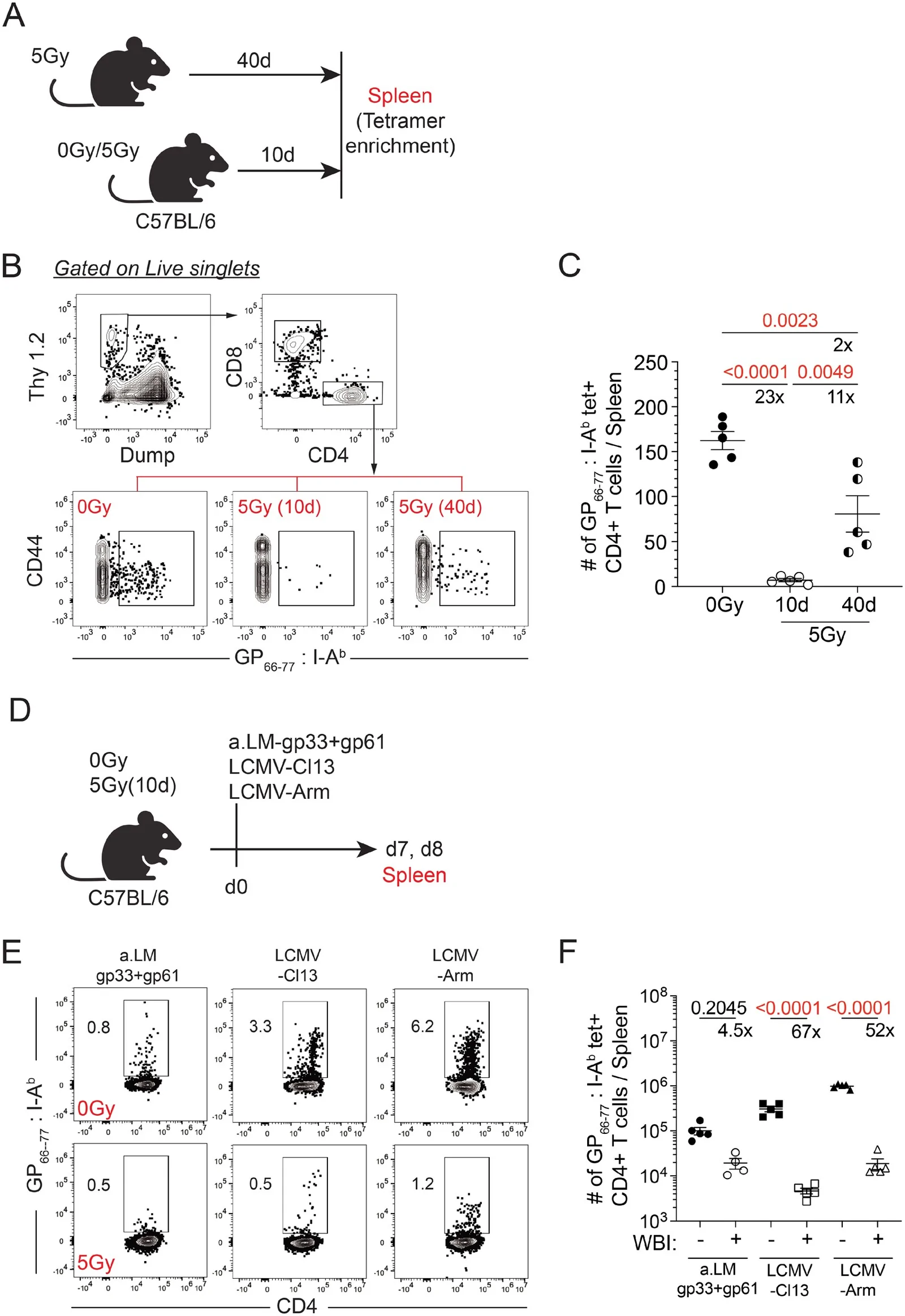
Diminished numbers of naïve CD4 T-cell precursors after WBI lead to poor effector CD4 T-cell expansion in response to intracellular infections. (A) Experimental design: Mice underwent 0 Gy or 5 Gy WBI either 40 or 10 d prior to euthanasia. Spleens were collected to enumerate GP_66-77_–specific CD4 T cells by tetramer enrichment. (B) Gating strategy and the representative flow plots of GP_66-77_–specific CD4 T cells. (C) Number of GP_66-77_–tetramer-specific CD4 T cells per spleen. (D) Experimental design: Mice were subjected to 0 Gy or 5 Gy WBI 10 d before infection with either 10^6^ CFU of a.LM-gp_33_+gp_61_; 2 × 10^6^ PFU of LCMV-Clone13; or 2 × 10^5^ PFU of LCMV-Armstrong (LCMV-Arm). Spleens were collected either 7 d after a.LM or 8 d after LCMV infection. (E) Representative flow plots of GP_66-77_–specific CD4 T cells in the spleen. (F) The number of GP_66-77_–specific CD4 T cells per spleen from 0 Gy or 5 Gy mice infected with different intracellular pathogens. The fold change between the groups is highlighted. *P* values were derived from 1-way ANOVA with Tukey’s multiple comparisons test (C) and Šidák’s multiple comparisons test (F). (C and F) Data are representative of 2 independent experiments with 4 to 5 mice per group. Graphs show the mean ± SEM. Graphical illustrations (A and D) are created with BioRender.com (http://biorender.com).

To determine whether epitope-specific naïve CD4 T cells exhibit similar numeric decline and recovery kinetics as total naïve CD4 T cells (Figs. 1H and 2C), we used pMHC-II tetramer enrichment^29–31^ to quantify CD4 T cells specific for LCMV-derived Ag (GP_66–77_) in the spleens of mice subjected to 0 Gy or 5 Gy WBI at 10 and 40 dpWBI (Fig. 3A). Magnetic enrichment of the tetramer-positive cells revealed a significant reduction in GP_66-77_–specific CD4 T-cell precursors at 10 dpWBI, followed by partial recovery by 40 dpWBI (Fig. 3B and C). These dynamics closely mirrored prior observations in the total naïve CD4 T-cell pool (Figs. 1H and 2C). Overall, these findings indicate that the post-WBI recovery kinetics of LCMV-derived, GP_66-77_–specific, naïve CD4 T-cell precursors are similar to that of the total naïve CD4 T-cell population.

To determine the extent to which WBI affects the host’s ability to mount Ag-specific CD4 T-cell responses to bacterial and viral infections, naïve B6 mice were subjected to either 0 Gy or 5 Gy WBI. Ten days later, when a significant decline in number of GP_66-77_–specific naïve CD4 T-cell precursors was observed (Fig. 3A–C), mice were infected with 1 of the following: an intracellular bacterium (a.LM expressing LCMV-derived epitopes gp_33_ and gp_61_ [a.LM-gp_33_+gp_61_]), or LCMV strains evoking chronic (LCMV-Cl13), or an acute (LCMV-Armstrong) virus infection (Fig. 3D).

Day 7 after a.LM and day 8 after LCMV (both Armstrong and Cl13) represent the peaks of effector CD4 T-cell expansion in young/naive B6 mice for the respective pathogens.^8^^,^^44–47^ At these time points, the number of GP_66-77_–specific effector CD4 T cells in the spleen was significantly lower in 5 Gy WBI mice compared with 0 Gy controls (Fig. 3E and F). After a.LM-gp_33_+gp_61_ challenge, the GP_66-77_–specific CD4 T-cell response in 5 Gy mice was 4.5-fold lower compared with the 0 Gy group. Similarly, responses to LCMV-Cl13 and LCMV-Armstrong strains were reduced by 67-fold and 52-fold, respectively, in irradiated mice (Fig. 3E and F). These findings suggest radiation-induced lymphopenia and the associated reduction in naïve CD4 T-cell precursors significantly impair the host’s ability to mount a robust, pathogen-specific CD4 T-cell expansion.

### WBI alters the number of virus-specific early memory CD4 T cells and impairs their cytokine production in response to cognate peptide stimulation

**Figure 4:**
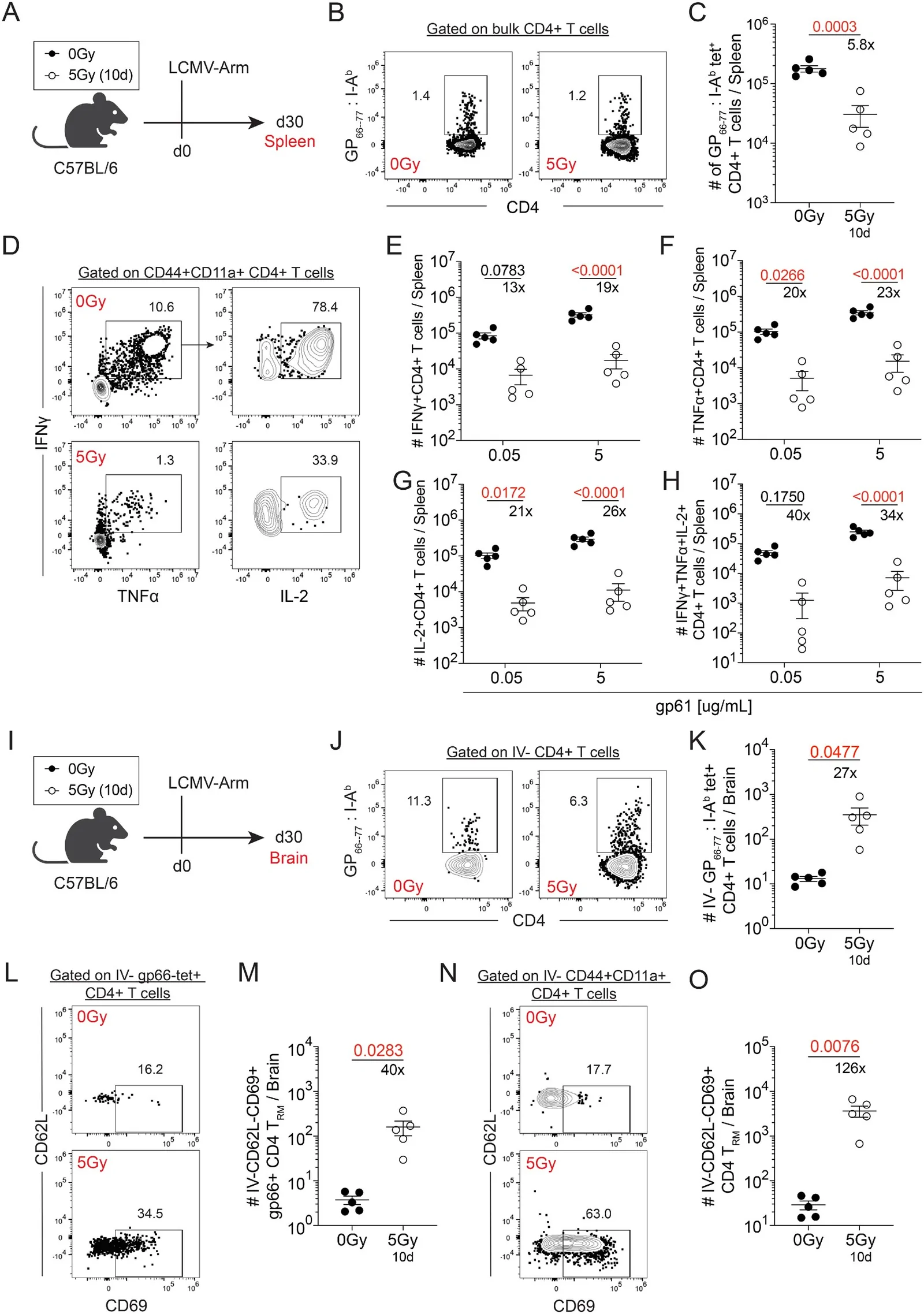
WBI-mediated CD4 T-cell intrinsic and extrinsic impairment influences the number, function, and migration potential of virus-specific early memory CD4 T cells. (A) Experimental design: Mice were subjected to 0 Gy or 5 Gy WBI 10 d before infection with LCMV-Armstrong (LCMV-ARM), and their spleens were collected after 30 d. (B and C) Representative flow plots of GP_66-77_–specific CD4 T cells in the spleen (B) and their absolute number (C). (D–H) Splenocytes harvested from mice in (A) were stimulated ex vivo with increasing concentrations of GP_61-80_ peptide in the presence of Golgi plug for 5 h, followed by intracellular immunolabeling for flow cytometry analyses. Representative flow plots of IFN-γ–, TNF-α–, and IL-2–producing effector CD4 T cells stimulated with the highest concentration of cognate peptide (D). (E–H) Absolute number of CD4 T cells producing IFN-γ (E), TNF-α (F), IL-2 (G), and all 3 cytokines (H) from the spleen of mice described in (A). (I) Experimental design: Mice were subjected to 0 or 5 Gy WBI 10 d before being infected with LCMV-Armstrong. Brains from these mice were harvested 30 d later. (J and K) Representative flow plots showing the i.v.^−^ GP_66-77_–specific CD4 T cells in the brain (J) and their absolute number (K). (L and M) Representative flow plots showing the i.v.^−^ GP_66-77_–specific CD4 T_RM_ (CD62L^−^CD69^+^) cells in brain (L) and their absolute number (M). (N and O) Representative flow plots showing the endogenous i.v. CD44^+^CD11a^+^ CD4 T_RM_ (CD62L^−^CD69^+^) cells in the brain (N) and their absolute number (O). Data are representative of 3 independent experiments with 5 mice per group. (C, G–J) Fold change between 0 Gy and 5 Gy(10 d) hosts is highlighted. (C, K, M, and O) *P* values were derived from an unpaired *t* test. (E–H) *P* values were derived from 2-way ANOVA with Šidák’s multiple comparisons test. Graphs show the mean ± SEM. Graphical illustrations (A and I) are created with BioRender.com (http://biorender.com).

The CD4 T-cell response to LCMV-Armstrong peaks at 8 d after infection (dpi), followed by contraction and transition into early-stage memory by 30 dpi.^44^^,^^45^^,^^48^^,^^49^ To determine whether the impaired pathogen-specific CD4 T-cell expansion observed in irradiated mice (Fig. 3D–F) leads to reduced memory CD4 T-cell generation, 0 Gy and 5 Gy WBI groups were analyzed at 30 dpi with LCMV-Armstrong (Fig. 4A). The number of GP_66-77_–specific, CD4 T cells remained 5.8-fold lower in the 5 Gy mice, indicating WBI impairs memory CD4 T-cell formation in response to infection (Fig. 4B and C). Notably, the reduction in GP_66-77_–specific CD4 T cells improved from a 52-fold decrease at 8 dpi (Fig. 3C) to a 5.8-fold decrease by 30 dpi (Fig. 4C), suggesting delayed or different kinetics of the pathogen-specific CD4 T-cell response that are initiated with diminished numbers of naïve Ag-specific CD4 T-cell precursors.

To determine whether memory CD4 T cells generated in irradiated hosts exhibit functional impairments, splenocytes were stimulated ex vivo with varying doses of the cognate GP_61-80_ peptide. The number of Ag-specific CD4 T cells producing effector cytokines (IFN-γ, TNF-α, and IL-2) was assessed (Fig. 4D). Importantly, cytokine-producing CD4 T-cell numbers were significantly reduced in a GP_61-80_ peptide dose-dependent manner in 5 Gy WBI mice compared with 0 Gy controls (Fig. 4E–G). Although single cytokine producers are important, polyfunctional T cells that produce multiple cytokines are more effective and play a crucial role in the ability to control viral infection.^50^^,^^51^ Strikingly, a 34-fold reduction in triple-positive (IFN-γ, TNF-α, and IL-2) CD4 T cells was observed in irradiated mice following stimulation with 5 μg/mL GP_61-80_ (Fig. 4H).

It is important to note that prior studies have highlighted that ex vivo stimulation with the GP_61-80_ peptide induces a polyclonal CD4 T-cell response, comprising both GP_66-77_ tetramer-positive and -negative CD4 T cells, both of which contribute to the effector cytokine response.^52^ Although not directly tested, comparing the magnitude of cytokine-producing cells (e.g., 19-fold and 34-fold reductions in Fig. 4G and J) with the reduction in tetramer-positive memory CD4 T cells (5.8-fold) (Fig. 4C) suggests that WBI leads to severe long-term impairment to both the quantity and per-cell functionality of memory CD4 T cells in response to infection.

### WBI-mediated CD4 T-cell intrinsic and extrinsic impairment influences their ability to differentiate into different memory T-cell subsets

Previous results (Figs. 3 and 4C) suggest radiation survivors have diminished ability to generate optimal numbers of memory CD4 T cells and point to the possibility that overall kinetics of Ag-specific CD4 T-cell responses (expansion, contraction, memory generation) might be influenced in mice exposed to WBI. Upon infection, naïve CD4 T cells typically differentiate into either T follicular helper (Tfh) cells or various Th cell subsets (eg Th1, Th2, Th9, Th17), depending on the pathogen and cytokine milieu.^4^^,^^53^ Intracellular pathogens such as LCMV-Armstrong preferentially generate Th1 and Tfh cells during the peak effector and memory stages.^44^^,^^45^^,^^48^^,^^49^ A recent study, using a.LM infection, demonstrated that CD4 T cells differentiate into 5 major subsets—2 Th1 and 3 Tfh subsets—at peak, contraction, and memory stages.^4^^,^^46^^,^^53^ Using flow cytometry, parabiosis, and single-cell RNA transcriptomic analysis, researchers on that study identified Ly6C^hi^ Th1 as effector memory T (T_EM_) cells, mantle zone–Tfh and pre-Tfh as central memory (T_CM_) cells, and Ly6C^lo^ Th1 and GC-Tfh as tissue-resident memory CD4 (T_RM_) T cells of their respective helper subsets.^46^

To test the extent to which WBI affects the composition of the memory CD4 T-cell pool, we examined these subsets 30 dpi with LCMV-Armstrong (Fig. S3A). Gating on GP_66-77_–specific CD4 T cells from the spleen revealed a significantly lower representation of the Ly6C^hi^ Th1 cells in irradiated mice (Fig. S3C). This is congruent with the poor pro-inflammatory cytokine response observed earlier (Fig. 4D–H). Although all 3 Tfh subset compositions remained unchanged, there was a minor decline in mantle zone–Tfh composition among WBI mice. Interestingly, the composition of Ly6C^lo^ Th1, the Th1 T_RM_ of the spleen, increased significantly in WBI mice (Fig. S3C). These data suggest prior radiation exposure alters the subset distribution of memory CD4 T cells after viral infection (Fig. S3C).

The significant increase in Th1 T_RM_ among GP_66-77_–specific CD4 T cells in the spleen (Fig. S3C) was intriguing and led us to explore WBI-mediated changes to CD4 T_RM_ cells in other nonlymphoid tissues. It has been previously demonstrated that ionizing radiation can disrupt barrier tissues throughout the body, including the blood–brain barrier (BBB).^54^ In non-irradiated mice, both neurotropic and non-neurotropic peripheral infections can induce CD4 and CD8 T_RM_ T cells in the brain.^55–58^ Given the brain’s central role in cognitive function, these cells serve as a reservoir for long-lived T_RM_ cells formed in response to various peripheral infections.^56^^,^^59^ These brain T_RM_ cells offer potent protection against subsequent intracranial infection, pointing to a functional role in mitigating infectious encephalitis. We asked, therefore, whether WBI-mediated BBB impairment facilitates the infiltration of CD4 T cells into the brain and formation of CD4 T_RM_ cells after LCMV-Armstrong infection.

To answer that question, we harvested the brains from 0 Gy and 5 Gy mice 30 d after LCMV-Armstrong infection (Fig. 4I). To distinguish circulating (i.v.^+^) from tissue-resident (i.v.^−^) immune cells, we used intravascular staining.^60^ At this early memory time point, we detected a 26-fold increase in i.v.^−^ GP_66-77_–specific CD4 T cells in the brains of 5 Gy mice compared with 0 Gy controls (Fig. 4J and K). More notably, there was a 40-fold increase in GP_66-77_–specific CD4 T_RM_ cells (i.v.^−^ CD62L^−^ CD69^+^) in the brains of 5 Gy mice (Fig. 4L and M), despite a 5.8-fold reduction of the same Ag-specific CD4 T-cell population in the spleen (Fig. 4B and C). Furthermore, we observed an even greater enrichment (126-fold) of endogenous CD4 T_RM_ cells (i.v.^−^ CD44^+^ CD11a^+^ CD62L^−^ CD69^+^) in the brains of 5 Gy mice (Fig. 4N and O). Even beyond 30 dpi (Fig. S4A), there was a 6.6-fold increase in i.v.^−^ GP_66-77_–specific CD4 T cells in the brain (Fig. S4F and G) of 5 Gy mice compared with the 0 Gy mice. At the same time, there was a 2.6-fold decrease in i.v.^−^ GP_66-77_–specific CD4 T cells in the salivary gland of 5 Gy mice compared with 0 Gy mice (Fig. S4D and E). This suggests the numeric increase in virus-specific CD4 T cells is unique to the brain and is not observed in other nonlymphoid tissues, such as the salivary gland. Together, these findings demonstrate that WBI-mediated BBB impairment enhances the infiltration of pathogen-specific memory CD4 T cells into the brain and significantly promotes the formation and enrichment of the brain-resident CD4 T_RM_ cell population.

### WBI limits naïve CD4 T-cell effector responses to self-antigens and diminishes the early onset of EAE

Because peripheral infection after WBI promotes virus-specific CD4 T-cell infiltration into the brain due to WBI-mediated BBB impairment (Fig. 4I–O), we wondered if WBI would promote neurological autoimmune diseases like MS. However, apart from BBB impairment, we can hypothesize that the naïve CD4 T-cell precursor frequency after WBI is also critical for effective expansion, contraction, and memory CD4 T-cell generation against self-antigens, as evidenced during infection. Therefore, we first tested if the myelin antigenic epitope MOG_38-49_–specific naïve CD4 T cells exhibited similar numeric decline and recovery kinetics as total naïve CD4 T cells (Figs. 1H and 2C). To that end, using the tetramer enrichment method described earlier, we determined the number of MOG_38-49_–specific naïve CD4 T cells in the spleens of mice subjected to 0 Gy or 5 Gy WBI at 10 and 40 dpWBI (Fig. S5A). Similar to GP_66-77_–specific naïve CD4 T-cell precursors (Fig. 3B and C), magnetic enrichment of the tetramer-positive cells revealed a significant reduction in MOG_38-49_–specific CD4 T-cell precursors at 10 dpWBI, followed by partial recovery by 40 dpWBI (Fig. S5B and C). These dynamics closely mirrored prior observations in the total naïve CD4 T-cell pool (Figs. 1H and 2C). Overall, these findings indicate that the post-WBI recovery kinetics of myelin-derived MOG_38-49_–specific naïve CD4 T-cell precursors are similar to that of the total naïve CD4 T-cell population.

**Figure 5:**
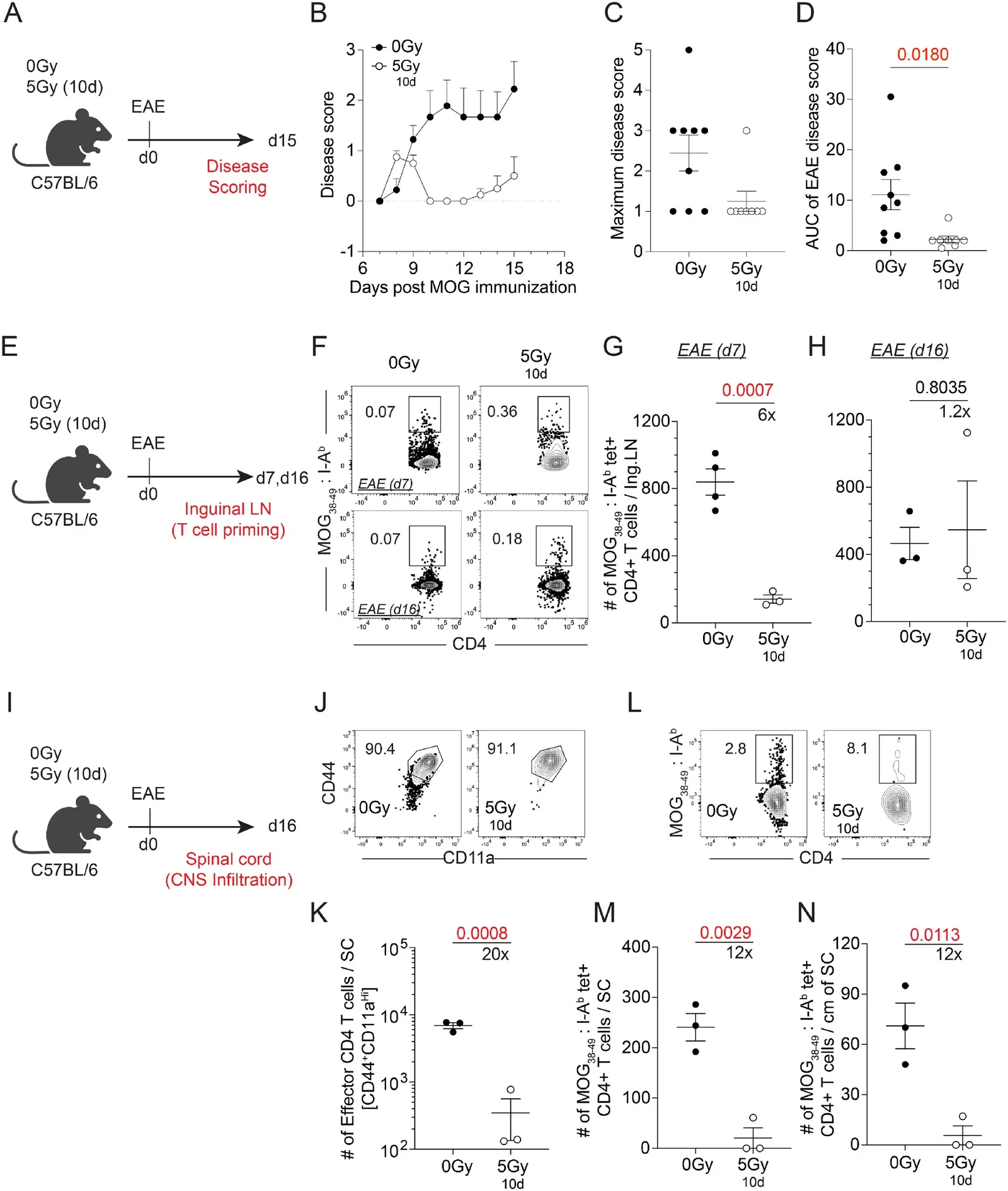
WBI limits naïve CD4 T-cell effector responses to self-antigens and diminishes the early onset of EAE. (A–D) Experimental design: Mice were subjected to 0 Gy or 5 Gy WBI 10 d before being immunized (s.c.) with MOG_35-55_ peptide (A), and their disease scores were monitored up to 15 d (B). Maximum disease score achieved by each mouse (C) and the area under the curve (AUC) from (B) is compared between 0 Gy and 5 Gy (10 d) mice (D). (E–H) At 7 and 16 dpi, Ing.LN were harvested (E). Representative flow plots of MOG_38-49_–specific CD4 T cells in the Ing.LNs (F), and their absolute number per Ing.LN 7 (G) and 16 dpi (H). (I–N) After 16 dpi, the SC was harvested from these mice (I). Representative flow plots of CD44^+^CD11a^+^ effector CD4 T cells infiltrating the SC (J), and their absolute number per SC from groups described in I (K). Representative flow plots of MOG_38–49_–specific CD4 T cells infiltrating the SC (L), and their absolute number per SC (M), or per centimeter of SC (N) from groups described in (I). Data are representative of 3 independent experiments with 8 to 9 mice (A–D) per group, or at least 3 mice (G–H, K, M–N) per group.(G–H, K, M–N) Fold changes between 0 Gy and 5 Gy(10 d) hosts are highlighted. (D–N) *P* values were derived from an unpaired *t* test. Graphs show the mean ± SEM. Graphical illustrations (A, E, and I) are created with BioRender.com (http://biorender.com).

As shown previously (Figs. 3 and 4, and Fig. S3), mice receiving 5 Gy WBI and infected with LCMV-Armstrong at 10 dpWBI had impaired CD4 T-cell effector expansion, memory formation, and per-cell cytokine production. Although an attenuated CD4 T-cell response to pathogens can be detrimental, a weakened response to self-antigens may be beneficial by mitigating autoimmune pathology. To assess how WBI influences naïve CD4 T-cell function in the context of autoimmunity, we used EAE, a widely used mouse model of MS. This model is extensively applied to study the immunopathobiology of MS, particularly the CD4 T-cell response to the MOG_35-55_ epitope of myelin in the CNS.^32^^,^^61^^,^^62^ Mice received 0 Gy or 5 Gy WBI and, 10 d later, were immunized with MOG_35-55_ emulsified in CFA on both flanks to induce EAE. PTX was administered i.p. on days 0 and 2 after immunization. This form of disease induction, termed “classical EAE,” primarily affects the SC.^63^ The mice were monitored over time using the standard clinical scoring system for classical EAE^33^ (Fig. 5A).

As expected, 0 Gy mice displayed progressive EAE symptoms (Fig. 5B). Interestingly, 5 Gy WBI mice immunized at 10 dpWBI had significantly attenuated clinical scores through at least day 15 after immunization (Fig. 5B). All mice in both groups reached a minimum clinical score of 1, confirming successful EAE induction (Fig. 5C). However, although greater than 50% of 0 Gy mice reached a score of ≥3, 90% of 5 Gy mice only reached a score of 1 (Fig. 5C). This difference in disease severity was reflected in a significantly lower area under the curve for the 5 Gy group (Fig. 5D).

We next asked whether the delayed disease onset was due to reduced priming of effector MOG_38-49_–specific CD4 T cells in the draining inguinal lymph node (Ing.LN) (Fig. 5E). At day 7 after immunization, prior to any overt EAE symptoms, we found that 5 Gy WBI mice had a 6-fold reduction in effector MOG_38-49_–specific CD4 T cells in the Ing.LN compared with 0 Gy controls (Fig. 5F and G). Interestingly, by day 16 after immunization, the number of effector MOG_38-49_–specific CD4 T cells in the Ing.LN had equalized between the 2 groups (Fig. 5H). These findings suggest a correlation between the reduced number of naïve MOG_38-49_–specific CD4 T-cell precursors and diminished early expansion of CD4 T cells after immunization in WBI-treated hosts. Additionally, consistent with observations following viral challenge, the kinetics of Ag-specific CD4 T-cell expansion—specifically, the timing of the peak effector response—may be delayed when initial priming occurs in a lymphopenic environment induced by WBI.

Because classical EAE involves CD4 T-cell infiltration into the CNS and subsequent demyelination of the SC, we next examined the extent of CD4 T-cell infiltration into the SC at day 16 after immunization. We quantified total effector CD4 T cells (CD44^+^CD11ahi) and MOG_38-49_–specific CD4 T cells in the SC (Fig. 5I). Consistent with the milder disease phenotype, we observed a 20-fold reduction in total effector CD4 T cells infiltrating the SC of 5 Gy WBI mice compared with 0 Gy controls (Fig. 5J and K). Moreover, there was a 12-fold reduction in MOG_38-49_–specific CD4 T cells in the SC of irradiated mice (Fig. 5L–N).

Taken together, these data demonstrate that WBI-induced lymphopenia impairs the priming and expansion of autoreactive CD4 T cells in secondary lymphoid organs, resulting in delayed disease onset and significantly reduced CNS infiltration during initial development of EAE.

### Time-dependent recovery of MOG_38-49_–specific CD4 T-cell precursors after WBI is associated with increased EAE severity upon immunization

**Figure 6:**
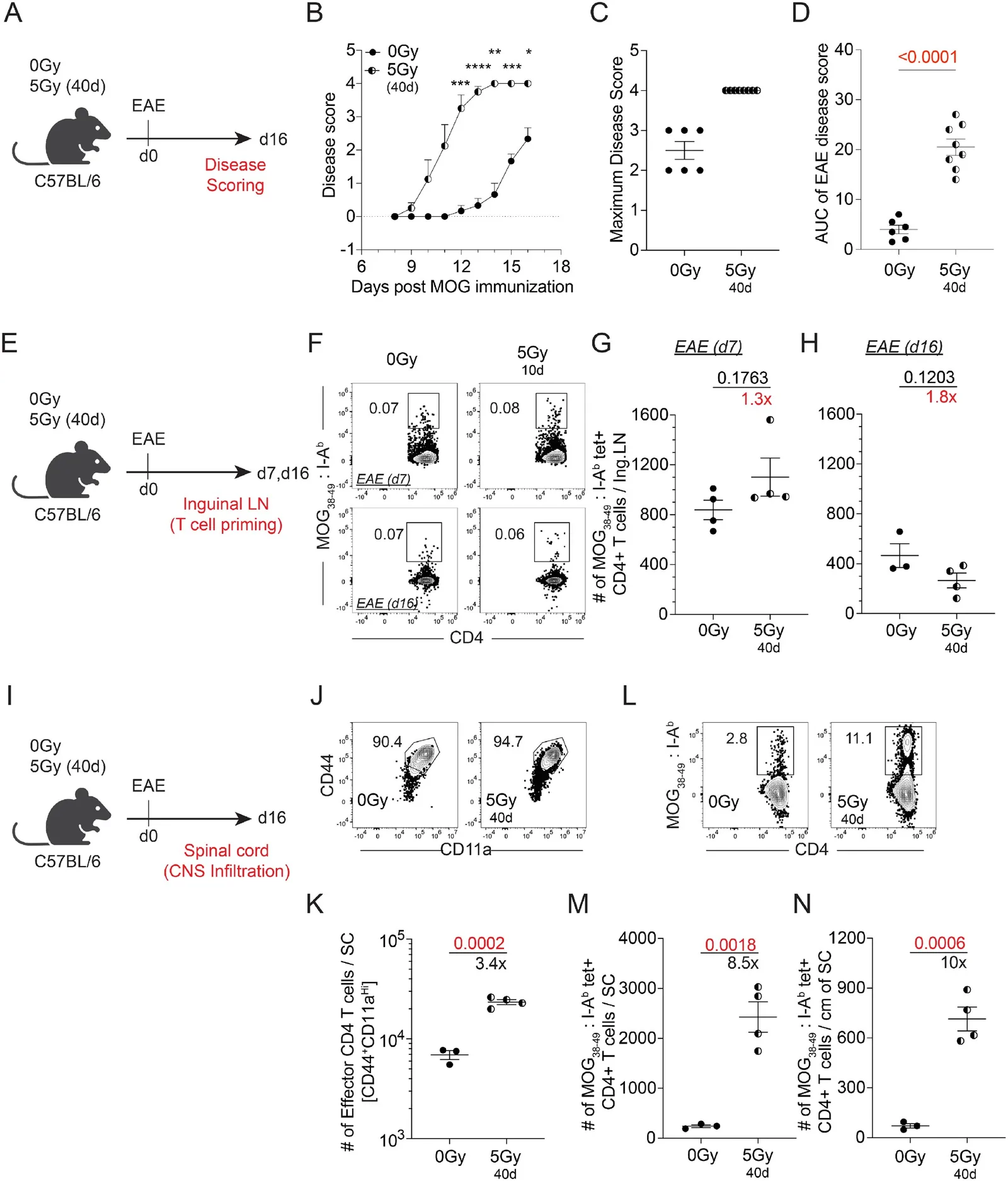
EAE disease is exacerbated in lymphoreplete WBI mice, evidenced by enhanced effector CD4 T-cell infiltration in the SC. (A–D) Experimental design: Mice were subjected to 0 Gy or 5 Gy WBI 40 d before being immunized (s.c.) with MOG_35-55_ peptide (A), and their disease scores were monitored up to 16 d (B). Maximum disease score achieved by each mouse (C) and the area under the curve (AUC) from (B) are compared between 0 Gy and 5 Gy (40 d) mice (D). (E–H) After 7 and 16 dpi, Ing.LN were harvested (E). (F–H) Representative flow plots of MOG_38-49_–specific CD4 T cells in the Ing.LNs (F), and their absolute number per Ing.LN 7 dpi (G) and 16 dpi (H). (I–N) At 16 dpi, the SC was harvested from these mice (I). Representative flow plots of CD44^+^CD11a^+^ effector CD4 T cells infiltrating the SC (J), and their absolute number per SC from groups described in I (K). Representative flow plots of MOG_38-49_–specific CD4 T cells from the SC (L), and their absolute number per SC (M) or per centimeter of SC (N) from groups described in (I). Data are representative of 3 independent experiments with 6 to 8 mice (A-D) per group, or at least 3 mice (G–H, K, M–N) per group.(G–H, K, M–N) Fold change between 0 Gy and 5 Gy (10 d) hosts are highlighted. (D–N) *P* values were derived from an unpaired *t* test. (B) Statistical significance derived from 2-way ANOVA with Šidák’s multiple comparisons test. *P ≤ 0.05, **P ≤ 0.01, ***P ≤ 0.001, and ****P ≤ 0.0001. Graphs show the mean ± SEM. Graphical illustrations (A, E, and I) are created with BioRender.com (http://biorender.com).

As shown earlier (Fig. S5), by 40 dpWBI, the number of naïve MOG_38-49_–specific CD4 T-cell precursors had nearly returned to levels observed in nonirradiated (0 Gy) mice. Therefore, we investigated whether delaying EAE induction until 40 dpWBI would affect the incidence and severity of EAE in 5 Gy WBI-treated mice (Fig. 6A). Interestingly, 5 Gy mice immunized at 40 dpWBI developed more severe EAE compared with 0 Gy controls (Fig. 6B–D). These irradiated mice exhibited higher average disease scores and earlier onset of clinical symptoms (Fig. 6B and D). Whereas only 50% of 0 Gy mice reached a maximum disease score of 3, all 5 Gy mice reached a score of 4 within 16 d of immunization (Fig. 6C).

To evaluate CD4 T-cell priming, we analyzed MOG_38-49_–specific CD4 T cells in the draining Ing.LN at both day 7 (before onset) and day 16 (after onset) after immunization. At both time points, the number of Ag-specific CD4 T cells was similar between 0 Gy and 5 Gy groups (Fig. 6E–H), indicating similar priming in both conditions. Interestingly, despite a similar number of effector CD4 T cells in the lymph nodes, 5 Gy mice immunized at 40 dpWBI had significantly increased infiltration of effector (CD44^+^CD11a^hi^) and MOG_38-49_–specific CD4 T cells into the SC at the prominent disease state (Fig. 6I–N). Overall, these findings indicate WBI-treated mice, once their encephalitogenic naïve CD4 T-cell precursors pool has recovered, exhibit exacerbated EAE upon immunization. This heightened disease severity is characterized by increased infiltration of pathogenic CD4 T cells into the SC.

The numeric increase in pathogenic CD4 T cells in the SC of WBI mice could also be driven by increased proliferation. To test that, we immunized 0 Gy mice and 5 Gy mice at 40 dpWBI with MOG_35-55_ and monitored disease progression for 15 dpi (Fig. S6A). As expected, the WBI mice progressed to a higher disease score (Fig. S6B), as evidenced by a 13-fold increase in MOG_35-55_–specific CD4 T cells in the SC compared with the 0 Gy mice (Fig. S6C and D). However, the expression of Ki67, a nuclear protein upregulated in proliferative cells, was similar between 0 Gy and lympho-replete mice (5 Gy, 40 dpWBI) (Fig. S6E and F). This suggests the increase in the number of pathogenic CD4 T cells observed in the SC of WBI mice is not driven by proliferation but through increased infiltration.

Although infiltration of T cells into the CNS is the first step, their functional potential to mediate neuroinflammation is key to causing disease severity. Data from the mouse model of infection clearly indicated that WBI impairs the cytokine production potential of virus-specific memory CD4 T cells (Fig. 4D–H). Therefore, we set out to determine the impact of WBI on the cytokine production potential of MOG-specific CD4 T cells infiltrating the SC (Fig. S6A). After 15 dpi, cells from the SCs of 2 mice with the same disease scores were pooled. These cells were incubated with Brefeldin A to limit cytokine release and then stained with MOG_38-49_–tetramer and intracellular cytokine markers. The frequency of MOG_38-49_–specific CD4 T cells producing the 2 key pro-inflammatory cytokines of EAE disease, IFN-γ and IL-17a, was similar between the 0 Gy and the lympho-replete (5 Gy, 40 dpWBI) mice (Fig. S6G and H). This phenotype was also observed among the activated (CD44^+^) CD4 T cells of SC (Fig. S6I and J). This suggests that the per-cell level of cytokine-producing potential is not impaired after WBI in the context of EAE, and shows that WBI does not affect the proliferation or neuroinflammatory potential of pathogenic CD4 T cells in the SC.

Because WBI does not affect the functional potential of encephalitogenic CD4 T cells, the numeric increase in these cells after WBI should be the key driver of disease severity. Our data show that 5 Gy mice immunized at 10 dpWBI progressed to lower disease scores, whereas those immunized at 40 dpWBI exhibited increased disease severity compared with 0 Gy controls. This dichotomy raises the possibility that 5 Gy mice immunized early after radiation exposure (10 dpWBI) may still develop EAE with delayed kinetics, influenced by the number of encephalitogenic MOG_38-49_–specific CD4 T cells (Fig. 5G and H). To test this hypothesis, we immunized 0 Gy mice and 5 Gy mice at either 10 or 40 dpWBI with MOG_35-55_ and monitored disease progression for 28 dpi (Fig. S7A). Consistent with previous observations, WBI delayed EAE onset in lymphopenic mice (5 Gy, 10 dpWBI) compared with 0 Gy controls, whereas it enhanced EAE severity in lympho-replete mice (5 Gy, 40 dpWBI) (Fig. S7B). Notably, because the number of MOG_38-49_–specific CD4 T cells gradually increased in 5 Gy mice immunized at 10 dpWBI (Fig. 5G and H), these mice eventually developed more severe disease, reaching similar scores as the 5 Gy, 40 dpWBI group (Fig. S7B). This enhanced disease phenotype persisted for up to 1 mo after immunization.

At 40 dpi, we analyzed MOG_38-49_–specific CD4 T cells in the SC and observed increased infiltration in 5 Gy mice, regardless of the timing of immunization (Fig. S7C). These findings confirm prior observations^64^ and establish a strong correlation between the number of encephalitogenic CD4 T cells and EAE development and severity. Moreover, they suggest radiation exposure alters CD4 T-cell–extrinsic environmental factors that contribute to disease potentiation in immunized WBI survivors.

### WBI-induced disruption of the BBB facilitates CD4 T-cell entry into the CNS and promotes EAE development

**Figure 7:**
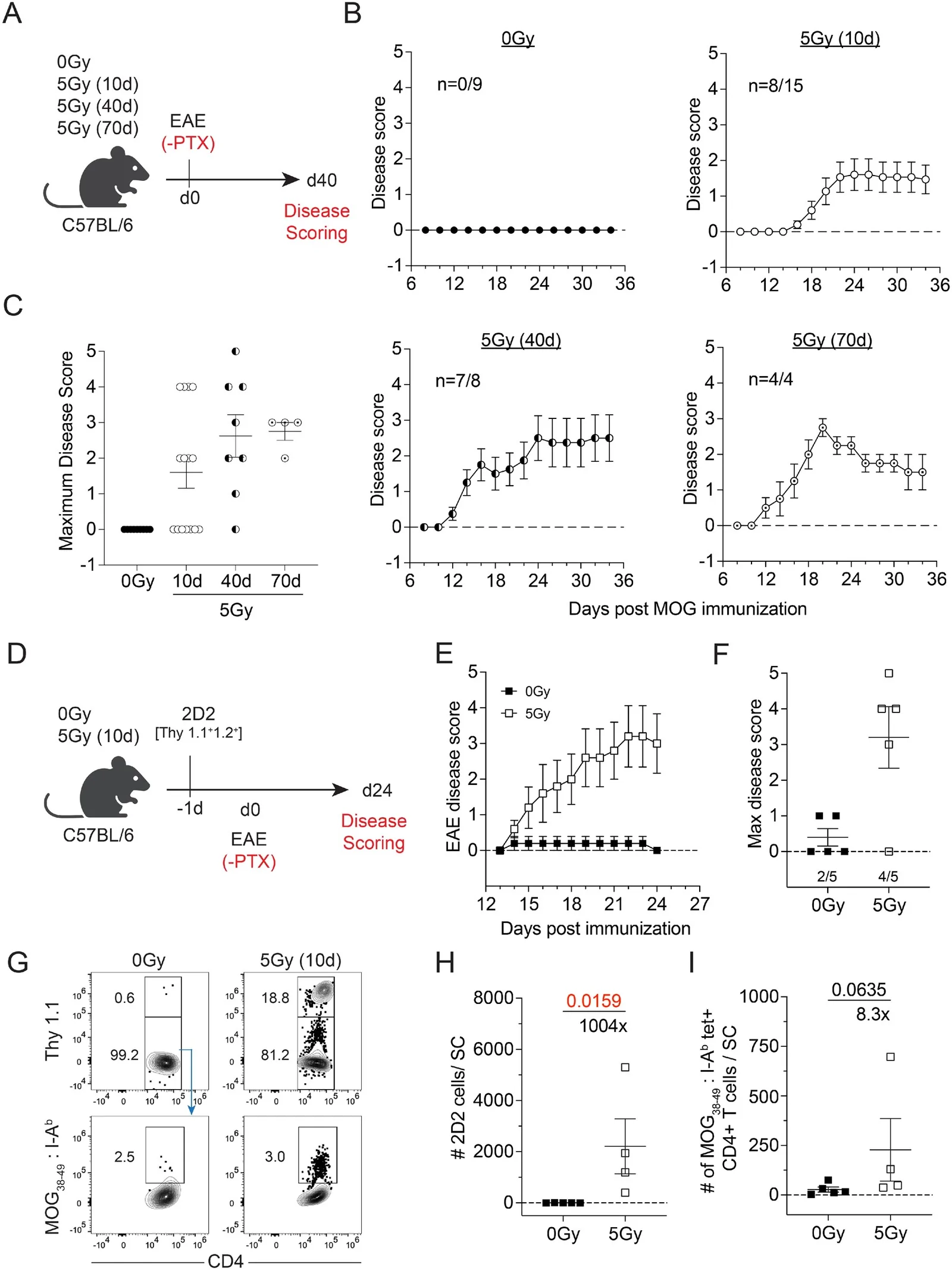
WBI-mediated impairment of the BBB and naïve CD4 T-cell homeostasis synergistically regulate EAE disease severity. (A–C) Experimental design: Mice were subjected to 0 Gy or 5 Gy WBI 10, 40, or 70 d before being immunized (s.c.) with MOG_35-55_ peptide without PTX administration (A). Disease scores were monitored up to 34 d after immunization (B). (C) The maximum disease score achieved by each mouse from (B) is compared between the groups described in (A). Data from 2 independent experiments are combined (A–C) with 4 to 15 mice per group. (D–I) Experimental design: Mice were subjected to 0 Gy or 5 Gy WBI and adoptively transferred naïve 2D2 cells after 9 dpWBI. The next day, these mice were immunized (s.c.) with the MOG_35-55_ peptide without PTX administration (D). Disease scores were monitored up to 24 dpi (E). The maximum disease score achieved by each mouse from € is compared between the groups described in D (F). Representative flow plots (G) and the number of pathogenic 2D2 CD4 T cells (CD90.2^+^) (H) and endogenous MOG_38-49_–specific CD4 T cells per SC (I). Data are representative of a single experiment (D–I) with at least 4 mice per group. (H and I) The fold change between 0 Gy and 5 Gy (10 d) hosts is highlighted. *P* values were derived from an unpaired *t* test. Graphs show the mean ± SEM. Graphical illustrations (A and D) are created with BioRender.com (http://biorender.com). Max, maximum.

In the mouse model of EAE used here, PTX administration is required to transiently open the BBB. Studies have shown that mice immunized with MOG+CFA in the absence of PTX do not generally develop EAE, with rare disease breakthroughs displaying delayed onset (>20 d) and clinical scores being very low.^65–68^ To determine whether WBI alters BBB permeability and permits easier access of encephalitogenic CD4 T cells into the SC, we immunized 0 Gy or 5 Gy mice at various time points after WBI (days 10, 40, and 70) with MOG_35-55_ plus CFA, but without PTX administration (Fig. 7A).

In the absence of PTX, none of the 0 Gy mice developed EAE, even 1 mo after immunization (Fig. 7B). Strikingly, 5 Gy mice developed EAE without PTX, regardless of the timing of immunization after WBI (Fig. 7B). Although 50% of mice immunized at 10 dpWBI developed disease, 90% of those immunized at 40 or 70 dpWBI developed moderate to severe EAE (Fig. 7C). These findings suggest WBI induces CD4 T-cell-extrinsic changes—including potential alterations to the BBB—that are sufficient to promote EAE development independently of PTX. To assess whether MOG_38-49_–specific CD4 T cells infiltrated the CNS in the absence of PTX, we harvested SC from mice 40 dpi. Compared with 0 Gy mice, which lacked detectable MOG_38-49_–specific CD4 T cells beyond levels seen in nonimmunized controls, all 5 Gy mice had increased numbers of MOG_38-49_–specific CD4 T cells in the SC (Fig. S8A–C). These data indicate WBI modifies BBB function in a way that facilitates the entry of MOG_38-49_–specific CD4 T cells into the CNS.

To directly assess WBI-induced BBB impairment, we i.v. administered FITC-labeled albumin into mice at 10 and 40 d after 0 or 5 Gy WBI. One hour later, brains were harvested and examined for FITC-albumin leakage using fluorescence microscopy. In the brainstem region—the junction between the brain and SC—the 0 Gy mice exhibited no FITC leakage (Fig. S8D, left). In contrast, 5 Gy mice had multiple regions of microleakage (Fig. S8D, middle and right), indicating compromised BBB integrity. This shows that WBI inherently impairs the BBB, facilitating increased infiltration of encephalitogenic CD4 T cells into the SC.

The data so far show that CD4 T-cell numeric recovery after WBI is essential for the development of EAE disease. However, both the CD4 T cells and the host are irradiated, making it hard to determine whether, after WBI, the CD4 T cells are functionally altered, or the environment is altered, or both. This can be tested by adoptive transfer of functional (nonirradiated) CD4 T cells into 5 Gy mice and immunizing them after 10 dpWBI. To that end, we adoptively transferred functionally potent 2D2 cells specific for the MOG_35-55_ epitope into 0 Gy and 5 Gy mice and immunized them the next day with MOG_35-55_ in the absence of PTX (Fig. 7D). The lymphopenic mice, in the presence of functional 2D2 cells, had higher disease scores of 3+ compared with 0 Gy controls (Fig. 7E and F). Interestingly, the number of functional pathogenic TCR-tg CD4 T cells was 1,004-fold higher, whereas that of the endogenous irradiated MOG_38-49_–tetramer-specific CD4 T cells was only 8.3-fold higher in 5 Gy hosts than in 0 Gy controls (Fig. 7G–I). This shows that lymphopenic irradiated hosts develop severe EAE if functional CD4 T cells are present, suggesting that both CD4 T-cell function and the environment are affected after irradiation. In summary, these results demonstrate WBI induces structural and functional changes in the BBB that, together with alterations in naïve CD4 T-cell homeostasis, synergistically regulate CD4 T-cell function and contribute to EAE pathogenesis.

## Discussion

Despite the increased risks of potential sub-lethal radiation exposure and the prevalence of standard RT procedures, the effects of radiation exposure on naïve CD4 T-cell maintenance and function remain largely unknown. Here, we reveal that WBI-induced CD4 T-cell dysfunction stems from both precursor depletion and extrinsic tissue damage that synergistically dictate their function. Although the effects of radiation exposure on naïve CD4 T-cell survival and maintenance have been explored, the functional responses of these cells after irradiation remain understudied at the host level. Using 2 different mouse models of intracellular infections and autoimmunity, we have demonstrated that host-level protection and immune pathology are dictated by the levels of CD4 T-cell precursors and by tissue damage after radiation exposure. These findings provide critical insights into future studies focused on vaccine regimen development and therapeutic considerations to protect patients undergoing RT from infections and neuroimmune disorders.

Existing studies addressing the effect of WBI on naïve CD4 T cells and their recovery were from the end of the 20th century.^69–71^ More recent studies have shown that targeted and sublethal WBI can affect the maintenance and function of resident and circulating memory CD8 T cells, respectively.^72^^,^^73^ However, the impact of WBI on CD4 T cells remains largely unexplored. Here, we demonstrate that WBI causes a severe decline in naïve CD4 T-cell numbers and that the thymus is critical for reconstituting the CD4 T-cell compartment. Our recent work showed alterations to the naïve CD8 TCR repertoire after WBI.^74^ Although our data indicate that self- and non–self-epitope-specific CD4 T cells undergo similar recovery kinetics after WBI, further studies are needed to clarify if there are differences in the recovery kinetics of CD4 T cells with different specificities. Furthermore, lymphopenic hosts undergoing thymic reconstitution generate effector-like CD8 virtual memory T (T_VM_) cells that share phenotypic and functional characteristics with bona fide true memory CD8 T cells.^74^^,^^75^ The T_VM_ equivalent of CD4 T cells is termed memory phenotype CD4 T cells, which recently have gained importance and exhibit a Th1-like phenotype and function.^76–78^ The proportion of the naïve CD4 T cells that share memory phenotype characteristics in the lympho-replete WBI mice and their functional contributions to the phenotype described in this study are unknown and will be interrogated in the future.

Although lympho-depletion is the intended outcome of HSCT procedures, the host-derived T cells subsequently reconstitute the T-cell compartment. HSCT studies in mice have revealed that HSCs transplanted into athymic mice lead to a higher ratio of donor- to host-derived T cells, whereas the ratio is significantly lower when transplanted into euthymic mice.^70^ This result suggests host thymus-derived T cells are a major contributor to the T-cell compartment in both WBI survivors and HSCT patients, revealing the functional potential of these newly educated CD4 T cells to protect the host. Human and mouse tumor studies have also shown that the lymphocyte function is impaired upon radiation exposure.^79–81^ Here, we demonstrate that the limited number of naïve CD4 T-cell precursors in the lymphopenic state after WBI limits their early effector responses against intracellular pathogens. Despite the initial deficit, these pathogen-specific CD4 T cells undergo modest expansion over time, suggesting a delayed effector and memory CD4 T-cell kinetics. The lower proportion of Th1 cells and the reduced production of pro-inflammatory cytokines among CD4 T cells from irradiated hosts suggest they may provide poor protection during secondary exposures. Further mechanistic studies could help identify therapeutic targets to improve CD4 T-cell responses against reinfection in irradiated hosts. Apart from mediating direct protection, memory CD4 T cells also confer protection indirectly by improving CD8 T-cell and B-cell responses.^6^^,^^17^^,^^82–84^ Building upon our findings, future studies could address the effect of WBI on the functional potential of specific memory CD4 T-cell subsets in mediating direct and indirect protection.

We previously demonstrated that immunizing mice early after sepsis-induced lymphopenia can delay the onset of EAE.^64^ Our findings are consistent with that, because the limited CD4 T-cell precursors at the lymphopenic state after WBI delayed EAE development. Interestingly, despite modest precursor recovery at the lymphoreplete state, irradiated mice developed a severe and rapid onset of EAE disease. This was also characterized by increased infiltration of MOG_38-49_–specific CD4 T cells in the SC. This result contrasts with previously published outcomes in a mouse model of sepsis in which immunizing mice at the lympho-replete state after sepsis delayed the onset of EAE.^64^ This outcome suggests WBI introduces a CD4 T-cell extrinsic effect that is potentially not recapitulated during sepsis. Our finding that the WBI-mediated BBB impairment was inherently capable of manifesting EAE disease in the absence of PTX suggests CD4 T-cell extrinsic defects from irradiation enhance EAE severity in lympho-replete hosts. Adoptive transfer of functional precursors into lymphopenic hosts led to severe EAE, suggesting that both CD4 T-cell intrinsic and extrinsic defects synergistically regulate EAE disease. Additional mechanistic studies addressing the effects of WBI on CD4 T-cell differentiation into inflammatory (Th1 and Th17) and regulatory CD4 T-cell subsets are needed to identify better interventions to limit EAE development in patients undergoing RT.

In mucosal tissues, CD4 T_RM_ cells generated during viral, bacterial, or fungal infections are protective.^85–87^ However, unlike CD8 T_RM_ cells, CD4 T_RM_ cells in other tissues are less explored, owing to CD4 T-cell and tissue-specific complexities.^4^ Recent elegant studies have highlighted that peripheral^57^^,^^59^ and direct neurological insults^55^ can establish both CD4 and CD8 T_RM_ cells in the CNS, which contribute to neuroinflammation. Self-epitope–specific T cells entering the CNS are known to be detrimental in the context of MS^88^; however, the consequences of increased infiltration of non–self-epitope-specific T cells into the brain remain unclear. Here, we set out to determine if WBI-mediated BBB impairment can enrich CD4 T_RM_ in the CNS of mice. We demonstrate that infecting a lymphopenic WBI host enhances CD4 T_RM_ enrichment specifically in the brain but not in the spleen or salivary gland. Whether CD4 T_RM_ enrichment after WBI directly contributes to enhanced baseline neuroinflammation and/or protection against pathogens warrants further investigation. Furthermore, as previously shown in other tissues,^89–91^ understanding whether these CD4 T_RM_ cells enriched in the brain after WBI are Th1- or Tfh-like can help researchers devise strategic vaccination approaches against neurotropic infections and brain tumors.

In summary, our findings reveal that CD4 T-cell dysfunction after WBI results from both cell-intrinsic precursor depletion and tissue-level BBB compromise. These aspects together manifest a poor CD4 T-cell functional response against pathogens and an exacerbated response against EAE. Because intentional or unintentional radiation exposure may broadly influence a host’s subsequent CD4 T-cell responses to tumors, pathogens, and self-antigens, this study offers previously undescribed impacts of the exposome on host adaptive immunity with relevance for medical countermeasures.

## Supplementary Material

vkag174_Supplementary_Data

## Data Availability

All data associated with this study can be found in the main text or the Supplementary Materials.
