# Supplementary material for: Whole-body irradiation causes long-term impairments in the maintenance and function of naïve CD4 T cells specific for self- and non-self-antigens
Source: J Immunol. 2026 Jul 13;215(7):vkag174. doi: 10.1093/jimmun/vkag174 (PMC13360226; doi:10.1093/jimmun/vkag174)
Supplement: vkag174_Supplementary_Data [file vkag174_supplementary_data.pdf]

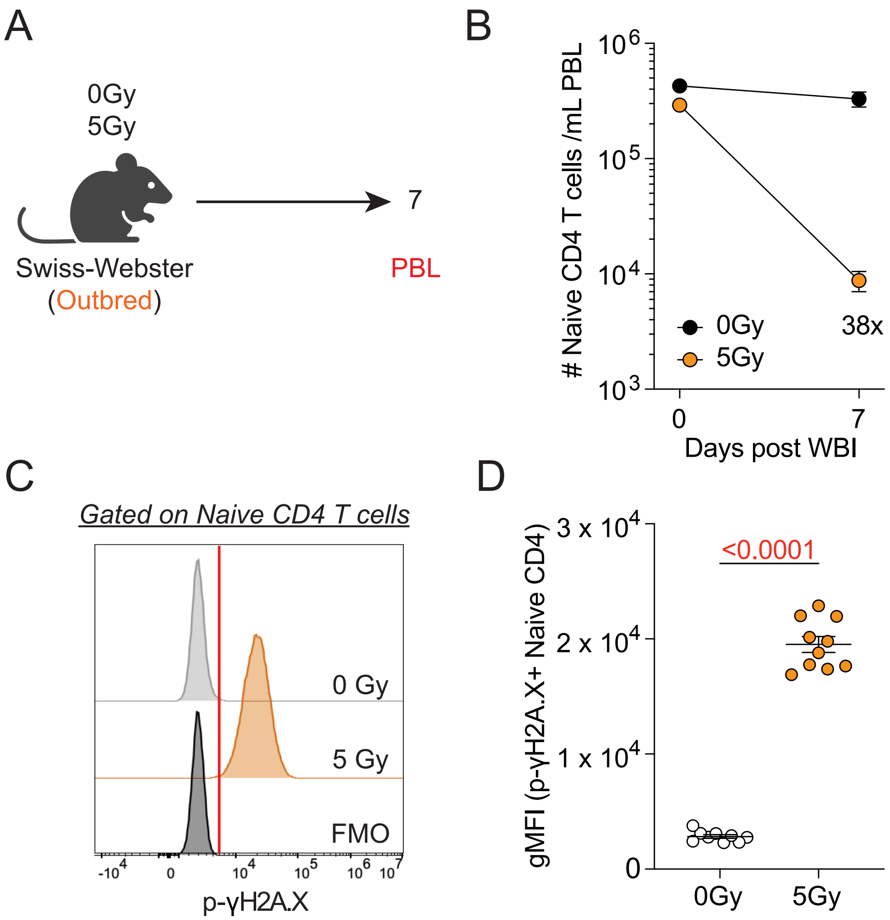

Figure S1. WBI-mediated DNA damage causes a severe numerical decline in naïve CD4 T cells of outbred mice. (A) Experimental design – Outbred Swiss-Webster mice were subjected to 0Gy or 5Gy WBI and monitored over time. (B) Number of CD4+CD44- naïve CD4 T cells per mL of blood 7 dpWBI. (C-D) Peripheral blood leukocytes were collected 3 hours after WBI. Representative flow plots (C) show the gMFI of p-γH2A.X expression (D) among naïve CD4 T cells (CD44-). Data are from a single experiment with 9 to 10 mice per group. In D, *P* values were derived from an unpaired t-test. Graphs show the mean ± SEM. Graphical illustrations (A) are created with BioRender.com (http://biorender.com).

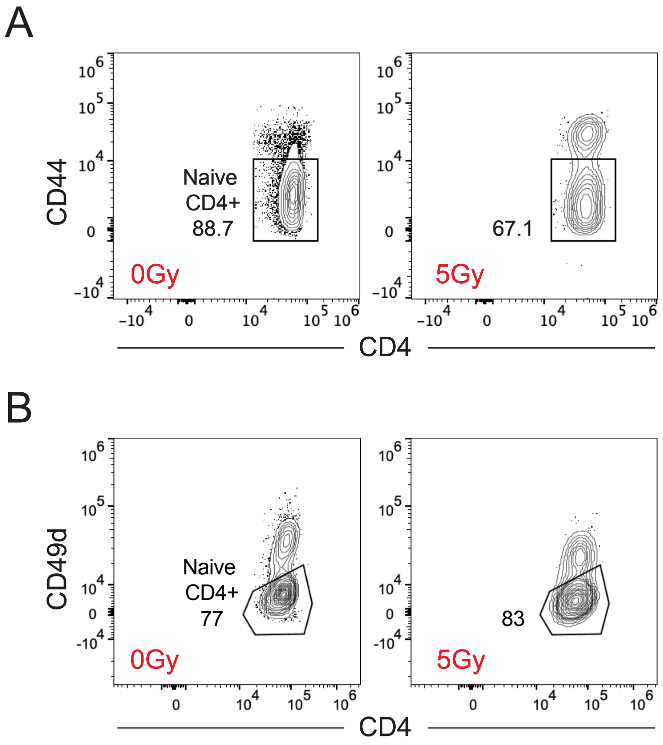

Figure S2. Representative flow plots show the gating strategy used to identify naïve CD4 T cells. After gating into CD4+CD8- singlets, CD44$^-$ (A) and CD49d$^-$ (B) gates were used to identify naïve CD4 T cells described in Figure 1G and 1H, respectively.

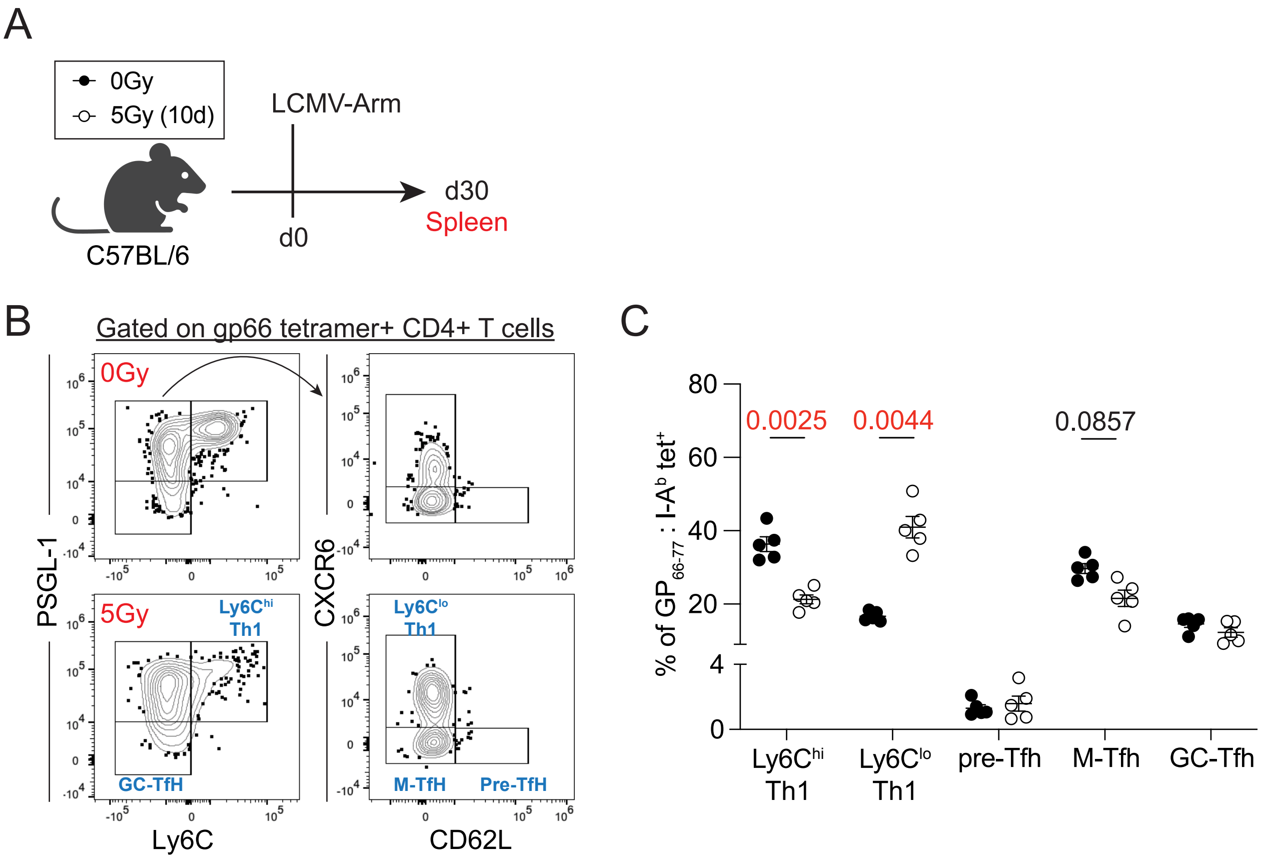

Figure S3. WBI alters the ability of early-memory CD4 T cells to differentiate into different memory T cell subsets. (A) Experimental design – Mice were subjected to 0Gy or 5Gy WBI 10 days before infection with LCMV-Armstrong, and their spleens were collected after 30 days. (B-C) Representative flow plots of memory CD4 T cell subsets of Th1 (Ly6C^{hi} and Ly6C^{lo}) and Tfh (Pre-Tfh, M-Tfh, and GC-Tfh) among $GP_{66\text{-}77}$-tetramer-specific CD4 T cells from mice described in A (D) and their percentages (E). Data are from a single experiment with 5 mice per group. *P* values (in C) were derived from two-way ANOVA with Sidak's multiple comparisons test. Graphs show the mean ± SEM. Graphical illustrations (A) are created with BioRender.com (http://biorender.com).

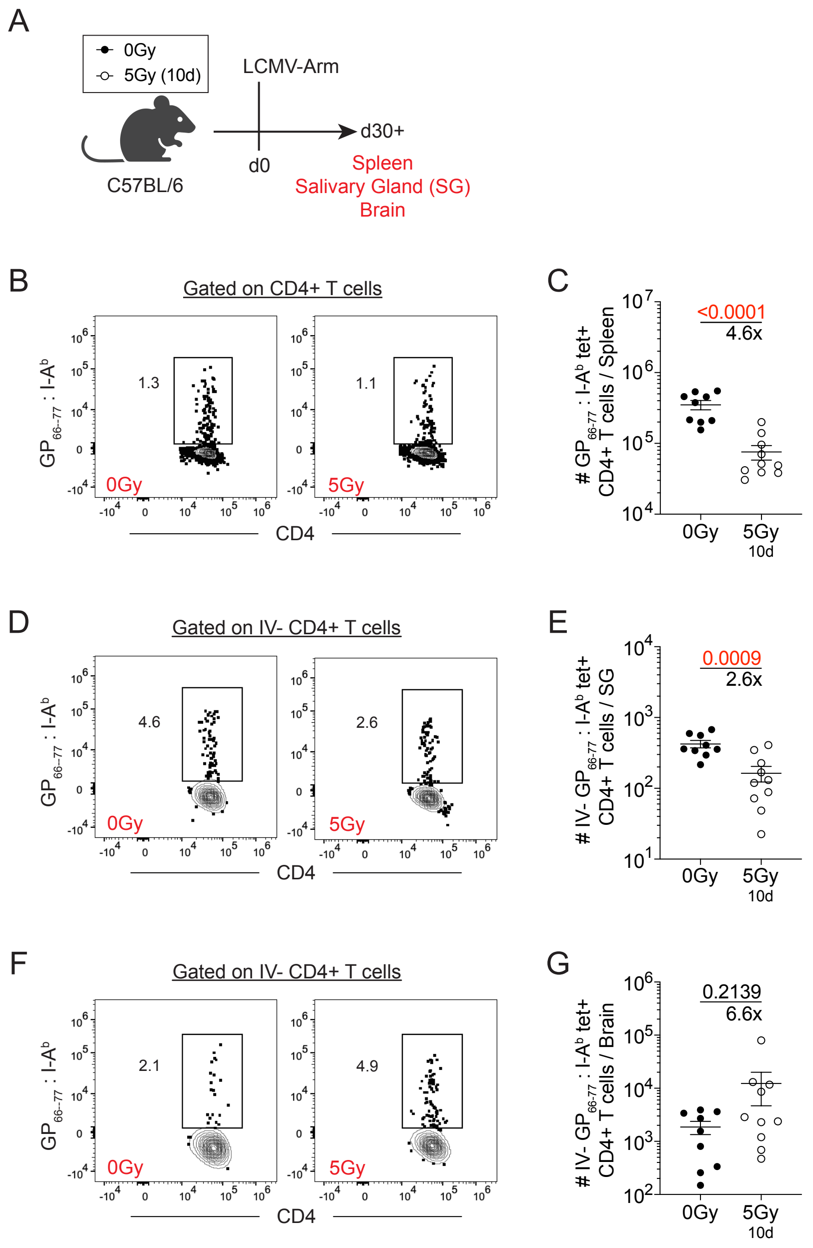

Figure S4. WBI-mediated increase in the number of virus-specific CD4 T cells is observed in the brain but not in the salivary gland. (A) Experimental design – Mice were subjected to 0Gy or 5Gy WBI 10 days before infection with LCMV-Armstrong, and their spleens were collected after 30 and 42 days. (B-C) Representative flow plots showing the $GP_{66\text{-}77}$-specific CD4 T cells in the Spleen (B) and their absolute number (C). (D-E) Representative flow plots showing the i.v.$^-$ $GP_{66\text{-}77}$-specific CD4 T cells in the salivary gland (SG) (D) and their absolute number (E). (F-G) Representative flow plots showing the i.v.$^-$ $GP_{66\text{-}77}$-specific CD4 T cells in the brain (SG) (D) and their absolute number (E). Data are a representative of 3 independent experiments. Within each group, data from both timepoints are pooled, yielding 9 to 10 mice per group. *P* values were derived from an unpaired t-test. The fold change between 0Gy and 5Gy(10d) hosts is highlighted. Graphs show the mean ± SEM. Graphical illustrations (A) are created with BioRender.com (http://biorender.com).

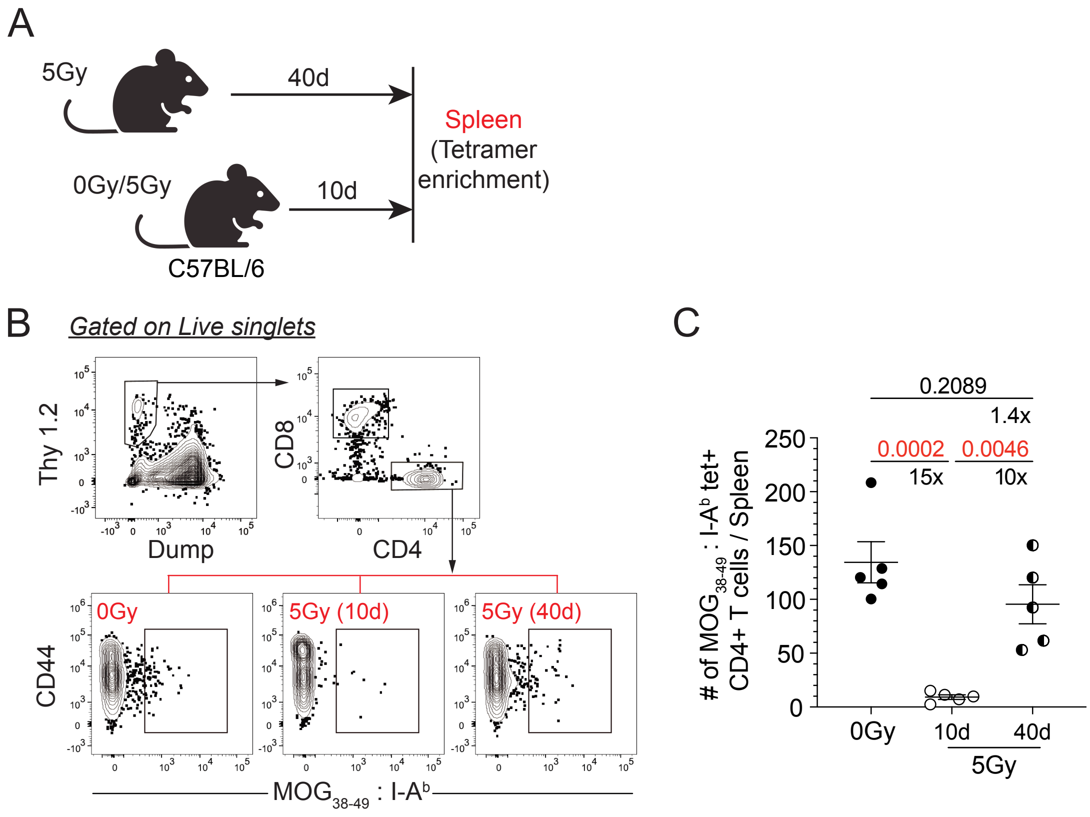

Figure S5. MOG$_{38-49}$-specific naïve CD4 T cell precursors undergo a similar decline and recovery as total naïve CD4 T cells following WBI. (A) Experimental design - Mice underwent 0Gy or 5Gy WBI either 40 or 10 days prior to euthanasia. Spleens were collected to enumerate MOG$_{38-49}$-specific CD4 T cells by tetramer enrichment. (B) Gating strategy and the representative flow plots of MOG$_{38-49}$-specific CD4 T cells. (C) Number of MOG$_{38-49}$-tetramer-specific naïve CD4 T cells per spleen. Fold change between the groups is highlighted. *P* values were derived from one-way ANOVA with Tukey's multiple comparisons test. Data derived from a single experiment with 5 mice per group. Graphs show the mean ± SEM, and *P* values of significance are highlighted in red text. Graphical illustrations (A) are created with BioRender.com (http://biorender.com).

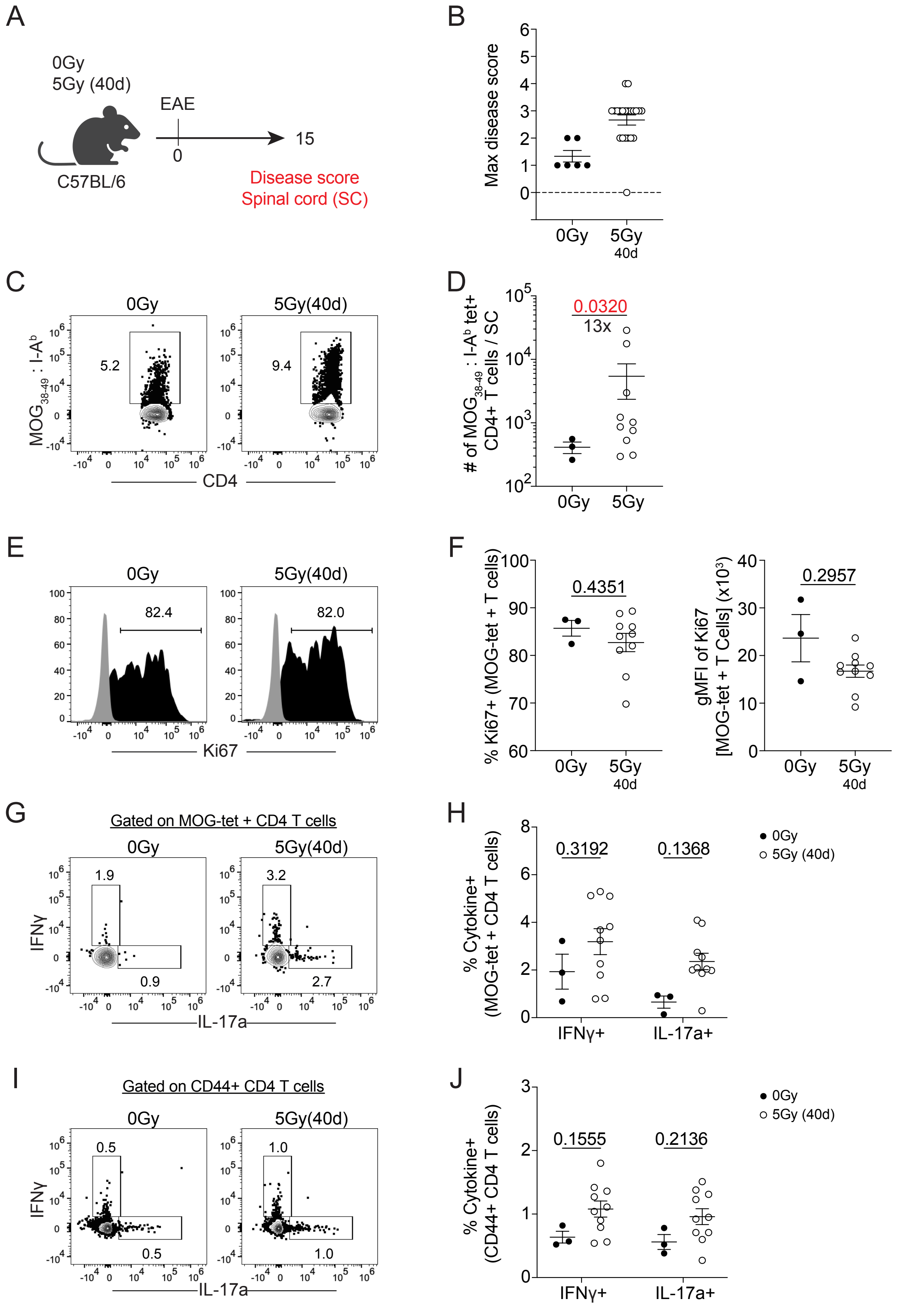
A
0Gy
5Gy (40d)
EAE
0
15
C57BL/6
Disease score
Spinal cord (SC)
B
Max disease score
0Gy
5Gy
40d
C
0Gy
5Gy(40d)
MOG38-49 : I-Ab
5.2
9.4
CD4
D
# of MOG38-49 : I-Ab tet+ CD4+ T cells / SC
0.0320
13x
0Gy
5Gy
E
0Gy
5Gy(40d)
82.4
82.0
Ki67
F
% Ki67+ (MOG-tet + T cells)
0.4351
0Gy
5Gy
40d
gMFI of Ki67 [MOG-tet + T Cells] (x10³)
0.2957
0Gy
5Gy
40d
G
Gated on MOG-tet + CD4 T cells
0Gy
5Gy(40d)
IFNγ
1.9
0.9
3.2
2.7
IL-17a
H
% Cytokine+ (MOG-tet + CD4 T cells)
0.3192
0.1368
IFNγ+
IL-17a+
0Gy
5Gy (40d)
I
Gated on CD44+ CD4 T cells
0Gy
5Gy(40d)
IFNγ
0.5
0.5
1.0
1.0
IL-17a
J
% Cytokine+ (CD44+ CD4 T cells)
0.1555
0.2136
IFNγ+
IL-17a+
0Gy
5Gy (40d)

Figure S6. WBI does not alter the proliferation and neuro-inflammatory cytokine production potential of encephalitogenic CD4 T cells in the spinal cord. (A) Experimental design - Mice were subjected to 0Gy or 5Gy WBI 40 days before being immunized (s.c.) with MOG_{35-55} peptide, and their disease scores were monitored up to 15 days. The maximum disease score achieved by each mouse from (A) is compared between 0Gy and 5Gy(10d) mice (B). (C-D) Representative flow plots of MOG_{38-49}-specific CD4 T cells infiltrating the SC (C), and their absolute number per SC (D). (E-F) Representative flow plots of Ki67 expression among MOG_{38-49}-specific CD4 T cells infiltrating the SC (E), and their frequency and gMFI (F). The grey histogram represents the Ki67-BUV737 FMO. (G-H) Representative flow plots of IFNγ and IL-17a expression among MOG_{38-49}-specific CD4 T cells infiltrating the SC (E), and their frequency (F). (I-J) Representative flow plots of IFNγ and IL-17a expression among $CD44^+$ CD4 T cells infiltrating the SC (I), and their frequency (J). Data are from a single experiment with 6 to 20 mice per group (A-B), in which tissue samples from 2 mice per group are pooled (C-J) before analysis, yielding 3 to 10 mice per group. In (G-H, K, M-N), fold change between 0Gy and 5Gy(10d) hosts are highlighted. *P* values in D-N were derived from an unpaired t-test. Graphs show the mean ± SEM. Graphical illustrations (A) are created with BioRender.com (http://biorender.com).

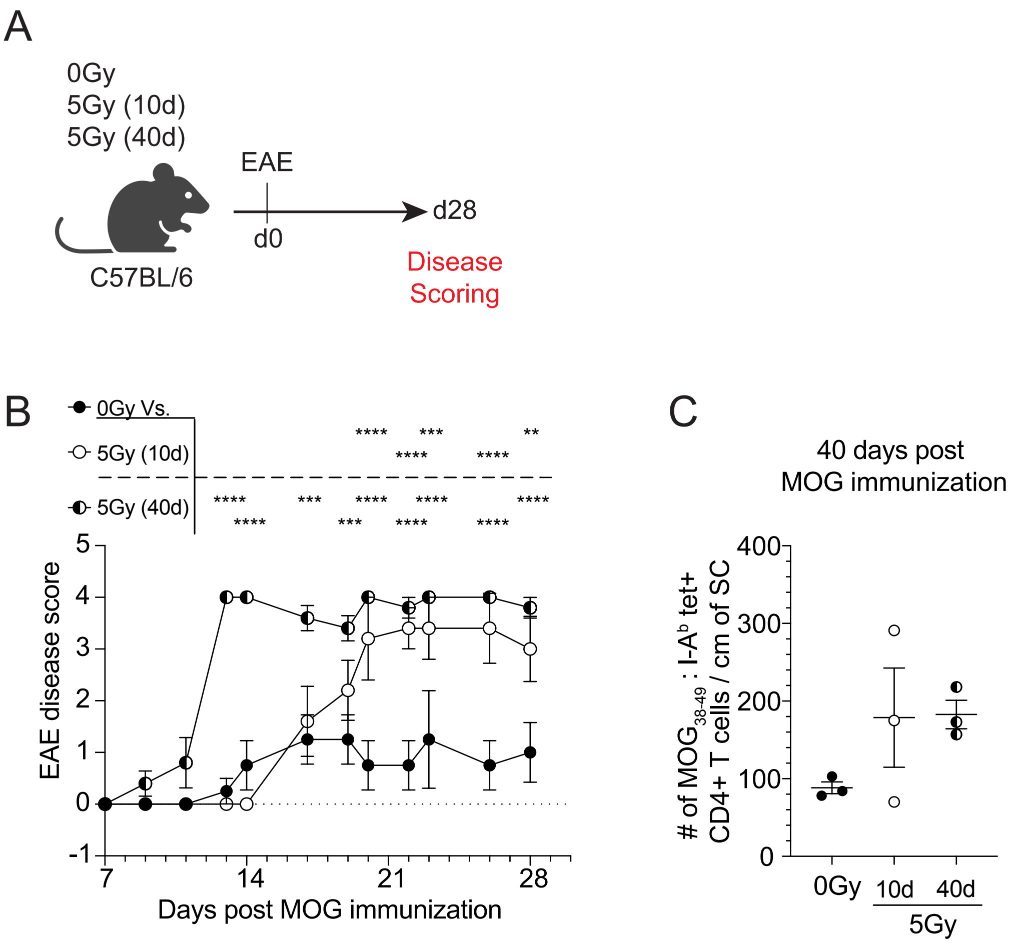

Figure S7. WBI delayed EAE onset in lymphopenic mice, as the number of $MOG_{38\text{-}49}$-specific CD4 T cells gradually increased compared to the 0Gy controls. (A) Experimental design - Mice were subjected to 0 or 5Gy WBI 10 or 40 days before being immunized (s.c.) with $MOG_{35\text{-}55}$ peptide +CFA + PTX (x2). (B) Disease scores were determined over the subsequent 28 days. At each time point, comparisons were made between 0 and 5Gy mice. (C) 40 days post-immunization, the absolute number of $MOG_{38\text{-}49}$-specific CD4 T cells per centimeter of SC was calculated from groups described in (S5.A). Data are from a single experiment with 4 to 5 mice (B) or 3 mice per group (C). Statistical significances were derived from two-way ANOVA with Dunnett's multiple comparisons test, where *p ≤ 0.05, **p ≤ 0.01, ***p ≤ 0.001, and ****p ≤ 0.0001. Graphs show the mean ± SEM. Graphical illustrations (A) are created with BioRender.com (http://biorender.com).

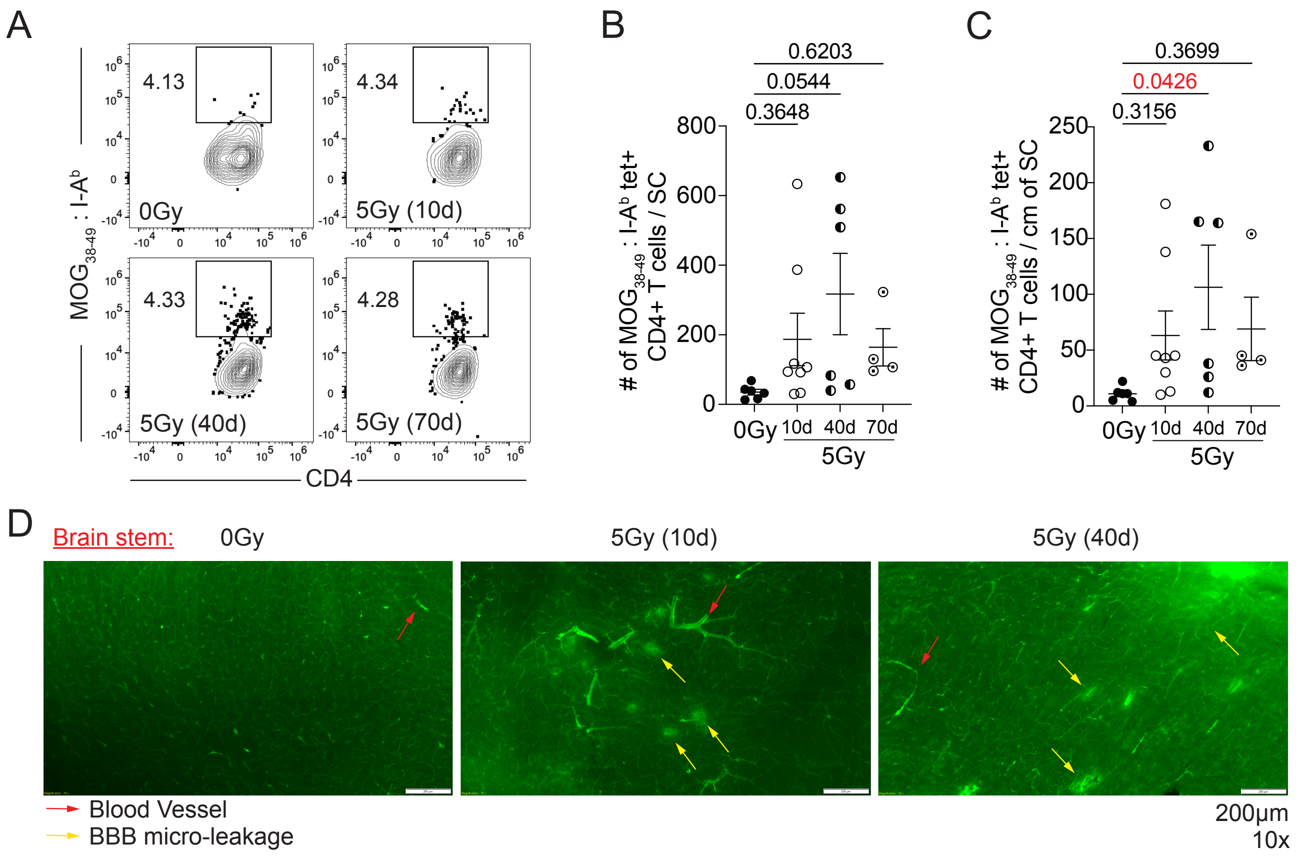

Figure S8. WBI inherently causes BBB impairment and increased infiltration of encephalitogenic CD4 T cells into the SC in the absence of PTX. (A-C) Representative flow plots of $MOG_{38\text{-}49}$-specific CD4 T cells from the SC 40 days post-immunization (A), their absolute number per SC (B), or number per centimeter of SC (C) from groups described in Figure 7A. Data are representative of 2 independent experiments with n=5-8 mice per group in each experiment. (D) Mice were subjected to 0Gy or 5Gy WBI 10 or 40 days before receiving FITC-albumin i.v., and their brain were harvested 60 minutes later. Agar-embedded tissues were sectioned and analyzed through fluorescence microscopy. Data derived from a single experiment with 5 mice per group. Representative images of the brainstem from each group are shown. *P* values in E-F were derived from one-way ANOVA with Dunnett's multiple comparison test. Graphs show the mean ± SEM.
